# Supplementary material for: Rhythm profiling using COFE reveals multi-omic circadian rhythms in human cancers in vivo
Source: PLoS Biol. 2025 May 27;23(5):e3003196. doi: 10.1371/journal.pbio.3003196 (PMC12136439; doi:10.1371/journal.pbio.3003196)
Supplement: S1 Text — (PDF) [file pbio.3003196.s002.pdf]

Supporting Information: Text

S1 Text Theoretical results

A Alternative formulation of sparse cyclic PCA

In this section, we motivate sparse cyclic PCA from a parameter estimation perspective. Consider a simple generative model for a feature j in the column-centered data matrix $\mathbf{X}$:

$$\mathbf{X}_{ij} = a_j \cos(\omega t_i) + b_j \sin(\omega t_i) + \varepsilon_{ij}, \;\; \varepsilon_{ij} \sim \mathcal{N}(0, \sigma^2), \; i = 1, \ldots N., \tag{S1}$$

where we have assumed that the noise variance is uniform across features for simplicity and $\omega = 2\pi/T$, where T is the period of the rhythm. A *rhythmic* feature j is defined by $(a_j, b_j) \neq (0,0)$ and an *arrhythmic* feature by

$$\mathbf{X}_{ij} = \varepsilon_{ij}, \;\; \varepsilon_{ij} \sim \mathcal{N}(0, \sigma^2), \; i = 1, \ldots N.$$

Eq. (S1) can be viewed as Fourier series approximation of a periodic signal at the fundamental frequency. The problem of determining the coefficients (a_j, b_j) in the model (S1) is often called cosinor regression in the circadian literature [25]. An important caveat is that in our setting, the sample times t_i are also unknown.

The log-likelihood of the data based on this generative model (S1) is:

$$L(\mathbf{X}) = \log\left[\prod_{j=1}^{p} \frac{1}{(2\pi\sigma^2)^{N/2}} \exp\left(-\frac{\|\mathbf{X}_{.j} - a_j \mathbf{u}_1 - b_j \mathbf{u}_2\|^2}{2\sigma^2}\right)\right], \tag{S2}$$

where $\mathbf{u}_1 = [\cos(\omega t_1), \ldots, \cos(\omega t_N)]^T$ and $\mathbf{u}_2 = [\sin(\omega t_1), \ldots, \sin(\omega t_N)]^T$. By definition, the elements of $\mathbf{u}_1, \mathbf{u}_2$ satisfy the circular constraint, ${\mathbf{u}_1}_i^2 + {\mathbf{u}_2}_i^2 = 1$. Letting $\mathbf{v}_1 = [a_1, a_2, \ldots, a_p]^T$ and $\mathbf{v}_2 = [b_1, b_2, \ldots, b_p]^T$, the log-likelihood (S2) can be rewritten as:

$$L(\mathbf{X}) = -\frac{Np}{2}\log(2\pi\sigma^2) - \frac{\sum_{j=1}^{p} \|\mathbf{X}_{.j} - a_j \mathbf{u}_1 - b_j \mathbf{u}_2\|^2}{2\sigma^2} = -\frac{Np}{2}\log(2\pi\sigma^2) - \frac{\|\mathbf{X} - \mathbf{u}_1\mathbf{v}_1^T - \mathbf{u}_2\mathbf{v}_2^T\|_F^2}{2\sigma^2}. \tag{S3}$$

The maximum likelihood estimate for the model parameters (a_j, b_j, t_i) is then equivalent to the rank-2 approximation introduced in (1)

$$\underset{\mathbf{u}_1, \mathbf{u}_2, \mathbf{v}_1, \mathbf{v}_2}{\text{argmin}} \; \|\mathbf{X} - d\mathbf{u}_1\mathbf{v}_1^T - d\mathbf{u}_2\mathbf{v}_2^T\|_F^2, \;\; {\mathbf{u}_1}_i^2 + {\mathbf{u}_2}_i^2 = 1, \;\; \|\mathbf{v}_1\|_2 = \|\mathbf{v}_2\|_2 = 1,$$

where w.l.o.g. we have introduced a parameter d to adapt the scale of the reconstruction after imposing an l_2-norm constraint on the $\mathbf{v}$s. To help identify the arrhythmic features, we encourage sparsity in $\mathbf{v}$ by enforcing a convex l_1-norm constraint ($\|\mathbf{v}_1\|_1 \leq s$, $\|\mathbf{v}_2\|_1 \leq s$). Ideally, we would like to enforce sparsity directly via a constraint on the l_0-norm, but since it is non-convex, following standard practice in the literature we relax it to an l_1-norm [47].

B Biconvexity of the optimization

Consider the optimized score in (3):

$$\max_{\mathbf{u}_1, \mathbf{v}_1, \mathbf{u}_1, \mathbf{v}_1, d} \left\{2d\mathbf{u}_1^T\mathbf{X}\mathbf{v}_1 + 2d\mathbf{u}_2^T\mathbf{X}\mathbf{v}_2 - d^2N\right\}$$
$$\text{s.t. } \|\mathbf{v}_1\|_2 = \|\mathbf{v}_2\|_2 = 1, \|\mathbf{v}_1\|_1 \leq s, \|\mathbf{v}_2\|_1 \leq s, \;\; \mathbf{u}_{1i}^2 + \mathbf{u}_{2i}^2 = 1, \; \forall i \in \{1, \ldots, N\}.$$

The objective function above is bilinear, i.e, linear in $\mathbf{u}$s for fixed $\mathbf{v}$s and vice versa. That is, the score can always be increased by scaling $\mathbf{u}$s and $\mathbf{v}$s by a factor larger than 1. Therefore, the solution to this problem remains unaltered when the constraints are expanded as follows:

$$\|\mathbf{v}_1\|_2 \leq 1,\ \|\mathbf{v}_2\|_2 \leq 1,\ \|\mathbf{v}_1\|_1 \leq s,\ \|\mathbf{v}_2\|_1 \leq s,\ \mathbf{u}_{1i}^2 + \mathbf{u}_{2i}^2 \leq 1,\ \forall i \in \{1, \ldots, N\}.$$

All the constraint sets are convex, since norms are convex and the intersection of convex sets is convex. Thus, convex constraint sets combined with a bilinear (biconvex) score proves the biconvexity of the optimization [46].
