# Supplementary material for: Rhythm profiling using COFE reveals multi-omic circadian rhythms in human cancers in vivo
Source: PLoS Biol. 2025 May 27;23(5):e3003196. doi: 10.1371/journal.pbio.3003196 (PMC12136439; doi:10.1371/journal.pbio.3003196)
Supplement: S2 Text — (PDF) [file pbio.3003196.s003.pdf]

S2 Text Pseudocode of sparse cyclic PCA

Algorithm 1 Sparse Cyclic PCA Algorithm

Input: $N \times p$ Data matrix $\mathbf{X}$, sparsity parameter s, tolerance tol, maximum iterations max_iter
Output: $\mathbf{v}_1, \mathbf{v}_2$ (sparse loading vectors), $\mathbf{u}_1, \mathbf{u}_2$ (cyclic principal components), d (scale factor)
Initialize $\mathbf{v}_1, \mathbf{v}_2$ with random draws of size $p \times 1$ from Laplace distribution
$\mathbf{v}_1 \leftarrow S(\mathbf{v}_1, \delta(s))$ // soft-threshold for sparsity constraint, Algorithm 2
$\mathbf{v}_2 \leftarrow S(\mathbf{v}_2, \delta(s))$ // soft-threshold for sparsity constraint, Algorithm 2
$\mathbf{v}_1 \leftarrow \mathbf{v}_1 / \|\mathbf{v}_1\|_2$ // l_2 normalize
$\mathbf{v}_2 \leftarrow \mathbf{v}_2 / \|\mathbf{v}_2\|_2$ // l_2 normalize
Initialize $\mathbf{u}_1 \leftarrow \cos(\phi)$ and $\mathbf{u}_2 \leftarrow \sin(\phi)$, where ϕ is random draw from uniform distribution $[0, 2\pi)$
$d \leftarrow \frac{(\mathbf{u}_1^T \mathbf{X}\mathbf{v}_1 + \mathbf{u}_2^T \mathbf{X}\mathbf{v}_2)}{N}$
$score \leftarrow 2d(\mathbf{u}_1^T \mathbf{X}\mathbf{v}_1 + \mathbf{u}_2^T X \mathbf{v}_2) - Nd^2$
$rss \leftarrow \|\mathbf{X}\|_F^2$
repeat
  // maximize **u**s keeping **v**s fixed
    $\mathbf{y}_1 \leftarrow \mathbf{X}\mathbf{v}_1$, $\mathbf{y}_2 \leftarrow \mathbf{X}\mathbf{v}_2$
    $\mathbf{y}_{circ} \leftarrow \sqrt{\mathbf{y}_1^2 + \mathbf{y}_2^2}$
    $\mathbf{u}_1 \leftarrow \mathbf{y}_1 / \mathbf{y}_{circ}$, $\mathbf{u}_2 \leftarrow \mathbf{y}_2 / \mathbf{y}_{circ}$
  // maximize **v**s keeping **u**s fixed
    $\mathbf{v}_1 \leftarrow S(X^T \mathbf{u}_1, \delta(s)) / \|S(X^T \mathbf{u}_1, \delta(s))\|_2$ // soft-threshold and normalize, Algorithm 2
    $\mathbf{v}_2 \leftarrow S(X^T \mathbf{u}_2, \delta(s)) / \|S(X^T \mathbf{u}_2, \delta(s))\|_2$ // soft-threshold and normalize, Algorithm 2
    $d \leftarrow \frac{(\mathbf{u}_1^T \mathbf{X}\mathbf{v}_1 + \mathbf{u}_2^T \mathbf{X}\mathbf{v}_2)}{N}$
    $score\_new \leftarrow 2d(\mathbf{u}_1^T \mathbf{X}\mathbf{v}_1 + \mathbf{u}_2^T \mathbf{X}\mathbf{v}_2) - Nd^2$
    $err \leftarrow \frac{|score\_new - score|}{|score|}$
    $rss \leftarrow \|\mathbf{X} - d(\mathbf{u}_1\mathbf{v}_1^T + \mathbf{u}_2\mathbf{v}_2^T)\|_F^2$
    $score \leftarrow score\_new$
until $interation\_count > max\_iter$ **or** $err \leq tol$
return $\mathbf{v}_1, \mathbf{v}_2$, $\mathbf{u}_1, \mathbf{u}_2$, d, rss

Algorithm 2 Soft-thresholding with L1 and L2 constraints

Input: $\mathbf{x}$ vector
Output: $\mathbf{x}$ vector constrained to satisfy l_1 and l_2 constraints
if $\|\mathbf{x}\|_1 / \|\mathbf{x}\|_2 \leq s$ **then**
  return $\mathbf{x}$ // No thresholding needed
else
  $\tilde{\mathbf{x}} \leftarrow \text{Sort}(|\mathbf{x}|, \text{decreasing})$
  // Algorithm 7 from [48]
  Define $\psi(c) \leftarrow \|S(\mathbf{x}, c)\|_1 / \|S(\mathbf{x}, c)\|_2$
  Find i such that $\psi(\tilde{\mathbf{x}}_i) < s < \psi(\tilde{\mathbf{x}}_{i+1})$
  Define $\delta \leftarrow \frac{\|S(x, \tilde{x}_i)\|_2}{i}\left(s\sqrt{\frac{i - \psi(\tilde{\mathbf{x}}_i)^2}{i - s^2}} - \psi(\tilde{x}_i)\right)$
  $s_{opt} \leftarrow \max(\tilde{\mathbf{x}}_i - \delta, 0)$
  return $S(\mathbf{x}, s_{opt})$
end if
