# Supplementary material for: Rhythm profiling using COFE reveals multi-omic circadian rhythms in human cancers in vivo
Source: PLoS Biol. 2025 May 27;23(5):e3003196. doi: 10.1371/journal.pbio.3003196 (PMC12136439; doi:10.1371/journal.pbio.3003196)
Supplement: S3 Text — (PDF) [file pbio.3003196.s004.pdf]

S3 Text Performance evaluation methodology

A Synthetic Datasets

To assess the performance of COFE, we generated simulated datasets, each of which is an $N \times p$ matrix with a fraction r of rhythmic features. The times at which the samples were collected, $t_1, \ldots, t_N$, the times COFE aims to predict, were chosen uniformly at random across one cycle of the underlying rhythm (one cycle spans T units of time). To include a wide range of non-sinusoidal rhythmic patterns in the data, the feature expression at each t_i was generated using periodic spline interpolation of 3 randomly chosen time-expression value pairs (see examples in S1A Fig.). Rhythmic features were standardized to have unit peak-to-trough amplitude and the arrhythmic features were assumed to be constant. All features were corrupted by additive white Gaussian noise $\mathcal{N}(0, \sigma^2)$. Thus, the SNR of the rhythmic features was $1/\sigma$. We generated synthetic datasets for combinatorial combinations of $N = 100, 200, 400$, $p = 2000$, $r = 0.1, 0.2, 0.33, 0.5$ and SNR=$0.5, 1.0, 2.0, 4.0$.

B Benchmark Datasets

We also analyzed the performance of COFE on time-series biological datasets (S1 Table) with the aim of comparing the predicted sample time-labels with true sample times.

C Metrics

We quantified two key aspects of the performance of COFE. COFE estimates the timing of the N samples within one cycle. These estimates are $\hat{t}_1, \ldots, \hat{t}_N \in [0, 1)$. To determine the reordering performance, we used median absolute position error (MAPE) defined as $\min_\psi \text{median}_{i=1,\ldots,N} \{|(\hat{t}_i - t_i/T - \psi) \mod 1|\}$ [5]. MAPE always lies in [0,0.5] and error as a fraction of the rhythm period. We can only determine the relative ordering of the samples within one cycle (hence we can estimate only $t_i/T \mod 1$) and not the absolute timing t_i of the samples (hence the search over different offsets ψ). As a rule of thumb, good performance is an MAPE less than 0.05, i.e., an error in timing reconstruction less than 5% of the rhythm period.

To quantify the ability of COFE to select exclusively rhythmic features to perform the reordering, we defined the fraction of features used for reordering that are rhythmic and the fraction of truly rhythmic biomarkers used for reordering. These are the ‘precision’ and ‘recall’ of the biomarker selection, respectively.
