# Supplementary material for: Rhythm profiling using COFE reveals multi-omic circadian rhythms in human cancers in vivo
Source: PLoS Biol. 2025 May 27;23(5):e3003196. doi: 10.1371/journal.pbio.3003196 (PMC12136439; doi:10.1371/journal.pbio.3003196)
Supplement: S4 Text — (PDF) [file pbio.3003196.s005.pdf]

S4 Text Analysis of TCGA data

A Data acquisition

Standardized (aligned and quantified) RNA-seq data, including from TCGA, was retrieved by means of recount3 (Bioconductor package v1.14.0)[51] annotated based on the GENCODE v29 (Ensemble 94). Unlike previous studies that attempted reordering of samples, we did not regress away any confounding variables and used raw data as is. We included only the histological types of each AC (Table S2) that included at least 250 samples. Moreover, we retained only a single tumor sample per patient per AC to train COFE (TCGA includes multiple tumor samples from some patients). We further kept protein-coding genes with an average read count of 64 reads/sample in the data. Gene expression was expressed in $\log_2$ cpm using DESeq2 (v1.40.2).

B COFE analysis

The primary analysis using COFE was performed using Python (S7 Table). The TCGA data was filtered to only keep genes (features) with mean $\log_2$-fold cpm of at least 7. COFE was run on all the 11 ACs individually with default parameters: 3-repeated 5-fold cross-validation with 5 restarts ($K = 5$, *repeats*=3, *restarts*=5). By default, COFE returns time-labels with respect to a normalized cycle in the interval [0, 1). We converted these time-labels to circadian time by multiplication with 24. Subsequent analyses and visualization was performed in R (S7 Table).

C Alignment of time-labels across ACs

The data-driven time-labels predicted by COFE are internally consistent, but the nature of the problem does not allow prediction of the absolute time-labels (the time reference is arbitrary) or the direction of time without additional information. We resolve this uncertainty by using the set of 15 expressed core clock genes to search for the consensus arrangement across all possible time references and directions of time in each AC. For this optimization, we expressed rhythmic gene expression of clock genes as phasors ($Ae^{i\phi}$) and used the complex SVD [12] to find the combination of time reference and direction for the ACs that maximized the explained variance. The arrangement that maximized this alignment was considered the consensus core clock peak time arrangement.

D Circadian parameters

Using the time-labels predicted by COFE (t_n for each sample n in the data $\mathbf{X}$), we have a complete time-series transcriptome and cancer-relevant proteome for each AC. We inferred circadian parameters from the time-series of feature p by fitting a cosinor function of the form $A_p \cos(\omega(t_n - t_p))$, $\omega = 2\pi/24$ [25], where A_p is the feature amplitude and t_p is the peak time of expression of feature p.
