# Supplementary material for: Rhythm profiling using COFE reveals multi-omic circadian rhythms in human cancers in vivo
Source: PLoS Biol. 2025 May 27;23(5):e3003196. doi: 10.1371/journal.pbio.3003196 (PMC12136439; doi:10.1371/journal.pbio.3003196)
Supplement: S1 Table — (PDF) [file pbio.3003196.s006.pdf]

Tissue	Sampling	Resolution	GEO/SRA Accession	Publication
mouse liver	independent	1h over 48h	SRP197108	Pan *et al.* [8]
human blood monocytes	longitudinal	3h over 42h		Wittenbrink *et al.* [9]
human dermis	longitudinal	4h over 24h	GSE205155	del Olmo *et al.* [10]
human epidermis	longitudinal	4h over 24h	GSE205155	del Olmo *et al.* [10]
malaria parasite	longitudinal	3h over 63-72h	GSE141653	Smith *et al.* [11]
