# Supplementary material for: Rhythm profiling using COFE reveals multi-omic circadian rhythms in human cancers in vivo
Source: PLoS Biol. 2025 May 27;23(5):e3003196. doi: 10.1371/journal.pbio.3003196 (PMC12136439; doi:10.1371/journal.pbio.3003196)
Supplement: S2 Table — (PDF) [file pbio.3003196.s007.pdf]

Adenocarcinoma	Histological Type
BRCA	Infiltrating Ductal Carcinoma
KIRC	Kidney Clear Cell Renal Carcinoma
PRAD	Prostate Adenocarcinoma Acinar Type
OV	Serous Cystadenocarcinoma
BLCA	Muscle invasive urothelial carcinoma (pT2 or above)
UCEC	Endometrioid endometrial adenocarcinoma
COAD	Colon Adenocarcinoma
THCA	Thyroid Papillary Carcinoma - Classical/usual
LIHC	Hepatocellular Carcinoma
LUAD	Lung Adenocarcinoma- Not Otherwise Specified (NOS)
KIRP	Kidney Papillary Renal Cell Carcinoma
