# Supplementary material for: Rhythm profiling using COFE reveals multi-omic circadian rhythms in human cancers in vivo
Source: PLoS Biol. 2025 May 27;23(5):e3003196. doi: 10.1371/journal.pbio.3003196 (PMC12136439; doi:10.1371/journal.pbio.3003196)
Supplement: S6 Table — (PDF) [file pbio.3003196.s011.pdf]

Adenocarcinoma	Best approximation error	Neglected Term	Neglected term as percentage of score
LUAD	3874048.762	1262.792	0.033%
PRAD	5371824.626	187.530	0.003%
LIHC	3580022.982	64.330	0.002%
COAD	4445436.789	1416.191	0.032%
BRCA	9587674.848	15.158	0.000%
UCEC	4899312.933	148.044	0.003%
OV	4835704.272	68.397	0.001%
KIRP	3407081.873	142.428	0.004%
BLCA	4561640.812	1074.322	0.024%
THCA	4186148.857	-1.499	0.000%
KIRC	6309507.411	1809.256	0.029%
