# Supplementary material for: Rhythm profiling using COFE reveals multi-omic circadian rhythms in human cancers in vivo
Source: PLoS Biol. 2025 May 27;23(5):e3003196. doi: 10.1371/journal.pbio.3003196 (PMC12136439; doi:10.1371/journal.pbio.3003196)
Supplement: S7 Table — (PDF) [file pbio.3003196.s012.pdf]

Language	Package name	Version
Python	python	3.9.15
	numpy	1.23.5
	pandas	1.5.2
	scipy	1.10.1
	seaborn	0.12.1
	joblib	1.2.0
	biocthings_client	0.2.6
R	Bioconductor	3.19
	biomaRt	2.60.1
	car	3.1-3
	CircStats	0.2-6
	clusterProfiler	4.12.6
	DESeq2	1.44.0
	dplyr	1.1.4
	effectsize	0.8.9
	ggnewscale	0.5.0
	ggpp	0.5.8-1
	ggraph	2.2.1
	ggrepel	0.9.6
	ggthemes	5.1.0
	msigdbr	7.5.1
	openxlsx	4.2.7.1
	org.Hs.eg.db	3.19.1
	patchwork	1.3.0
	PCAtools	2.16.0
	ReactomePA	1.48.0
	reactome.db	1.88.0
	recount3	1.14.0
	reticulate	1.39.0
	SuperExactTest	1.1.0
	tidygraph	1.3.1
	tidyverse	2.0.0
	UpSetR	1.4.0
