# Supplementary figures and images for: Rhythm profiling using COFE reveals multi-omic circadian rhythms in human cancers in vivo
Source: PLoS Biol. 2025 May 27;23(5):e3003196. doi: 10.1371/journal.pbio.3003196 (PMC12136439; doi:10.1371/journal.pbio.3003196)

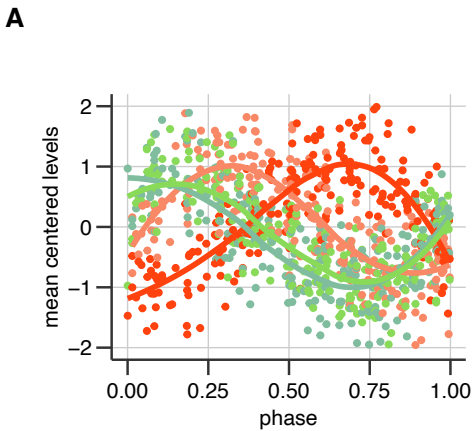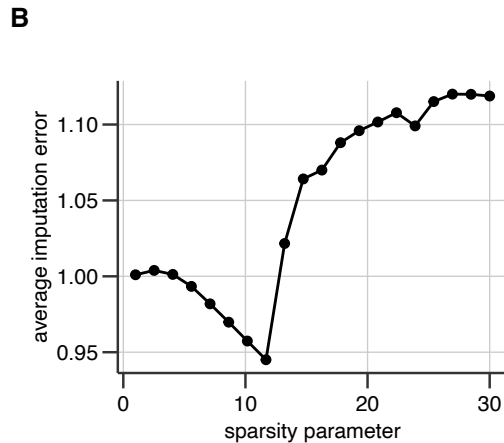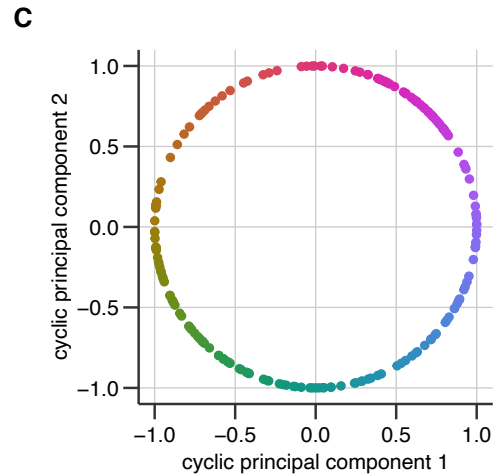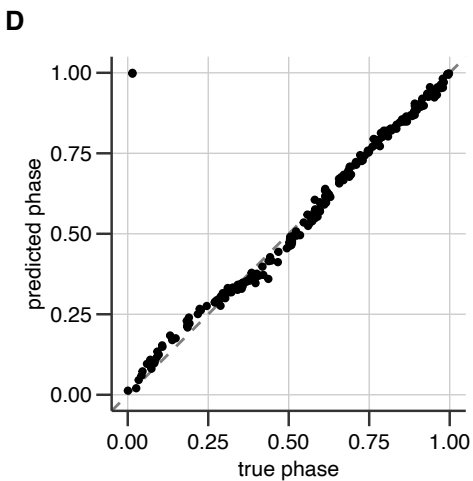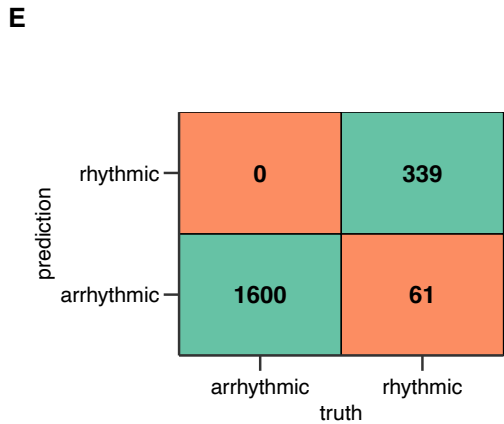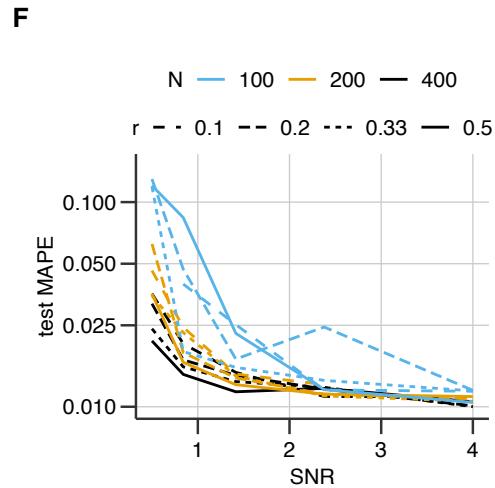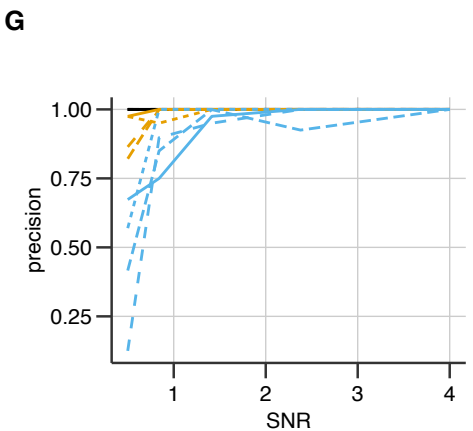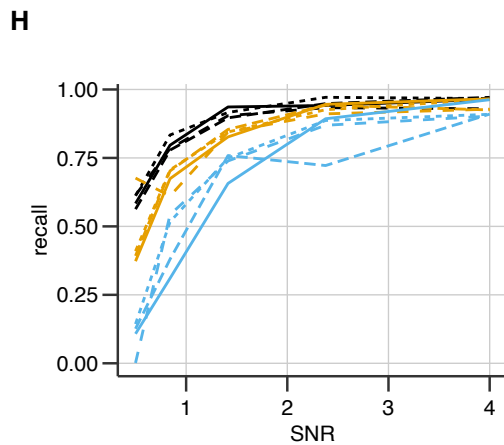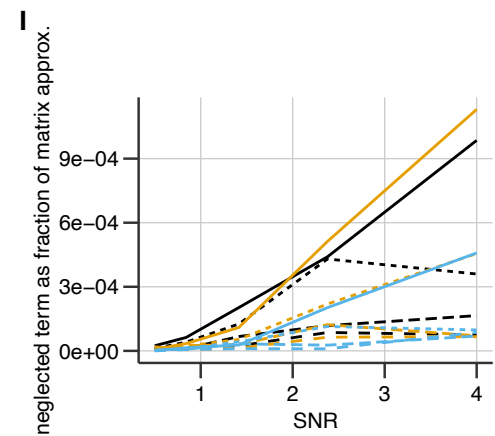

Supplement: S1 Fig — (A–E) A typical COFE run on an example data set with N = 2000 features, of which 400 were rhythmic. (A) Raw data for a few rhythmic features. (B) The output of repeated 5-fold cross-validation. (C) The underlying circular manifold reconstructed by COFE. COFE outputs the reconstructed phase within a cycle for each sample (D) and identifies rhythmic features in the data (E). (F) Temporal ordering performance of COFE measured using median absolute position error (MAPE) on data with p = 2000 features and different signal-to-noise ratios (SNR), number of samples (N) and fraction of rhythmic features (r). Precision (G) and recall (H) performance for rhythmic feature identification for the same synthetic data in (F). (I) The ratio of the cross term dropped from Eq 2 as a fraction of the matrix approximation error for the synthetic data in (F). The data underlying Figure panels can be found in S1 Data. (PDF) [file pbio.3003196.s013.pdf]

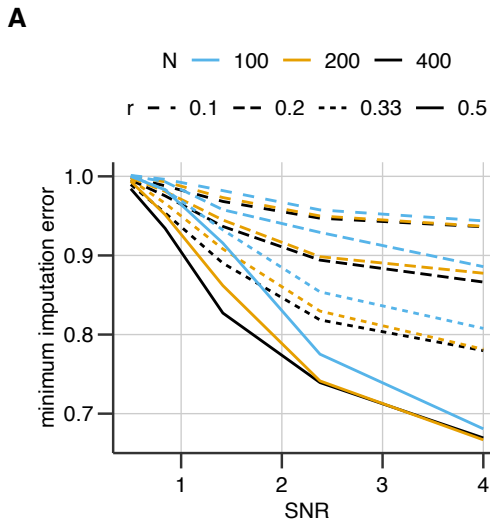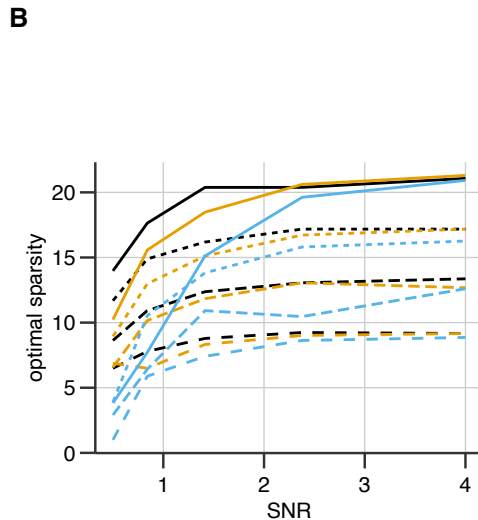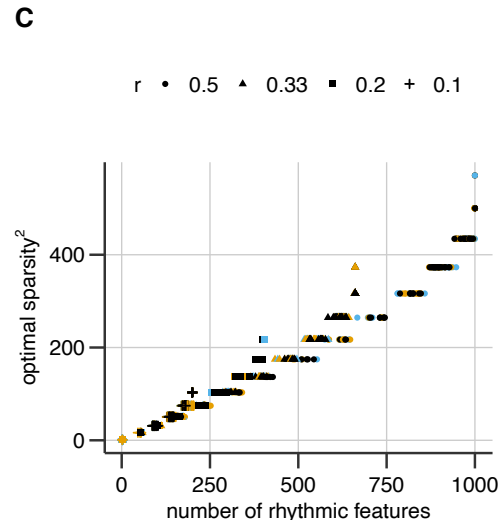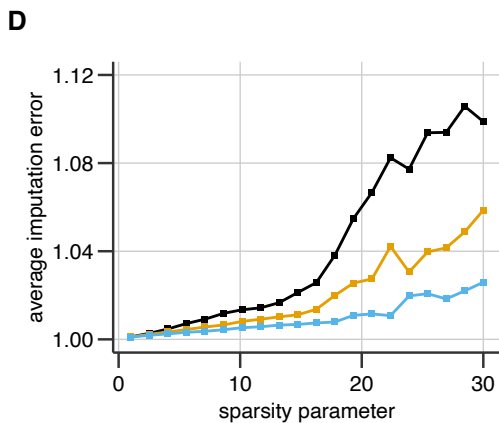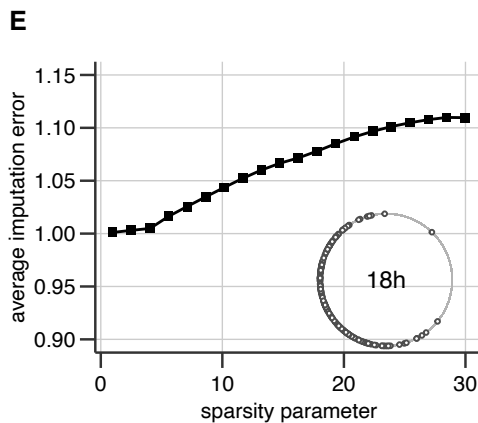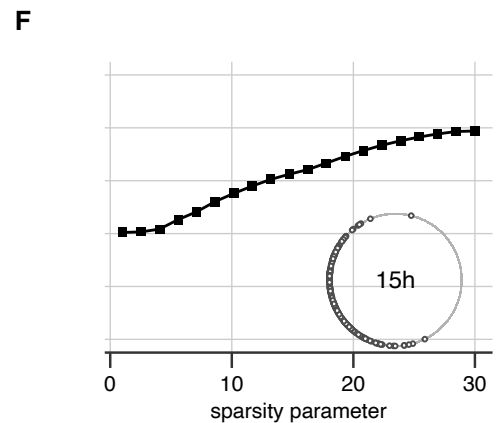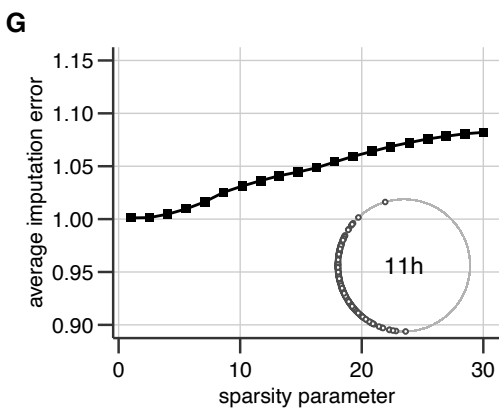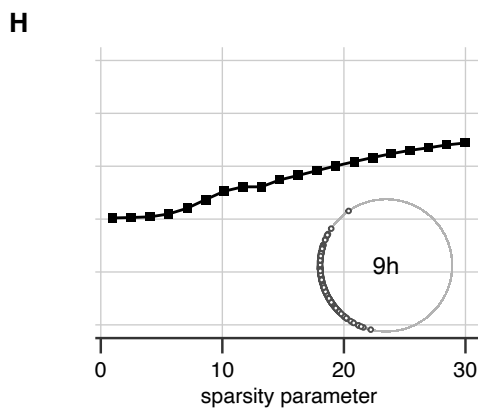

Supplement: S2 Fig — The minimum imputation error (A) and optimal sparsity parameter (B) during CV for different combinations of signal-to-noise ratios (SNR), number of samples (N) and fraction of rhythmic features (r) in S1F Fig. (C) The relationship between the optimal sparsity parameter and the number of identified rhythmic features. (D) Output of repeated-5-fold CV for a synthetic dataset with only arrhythmic features (2000) for different number of samples (N). (E-H) Output of repeated-5-fold CV on datasets with 200 samples that were restricted to different fractions of the 24h cycle (see inset), and 2000 features, of which 400 were rhythmic. The data underlying Figure panels can be found in S1 Data. (PDF) [file pbio.3003196.s014.pdf]

**A**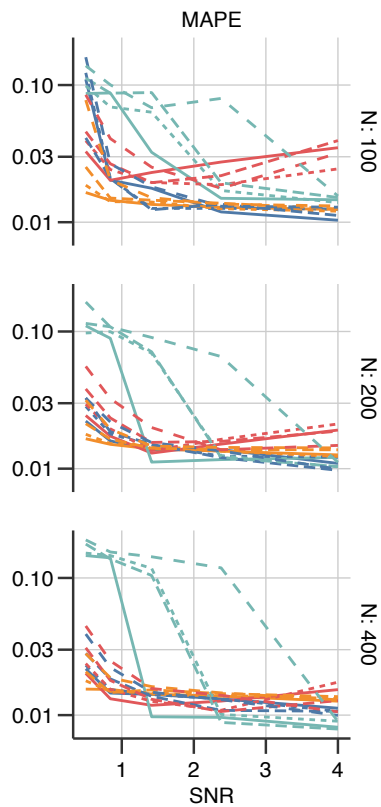**B**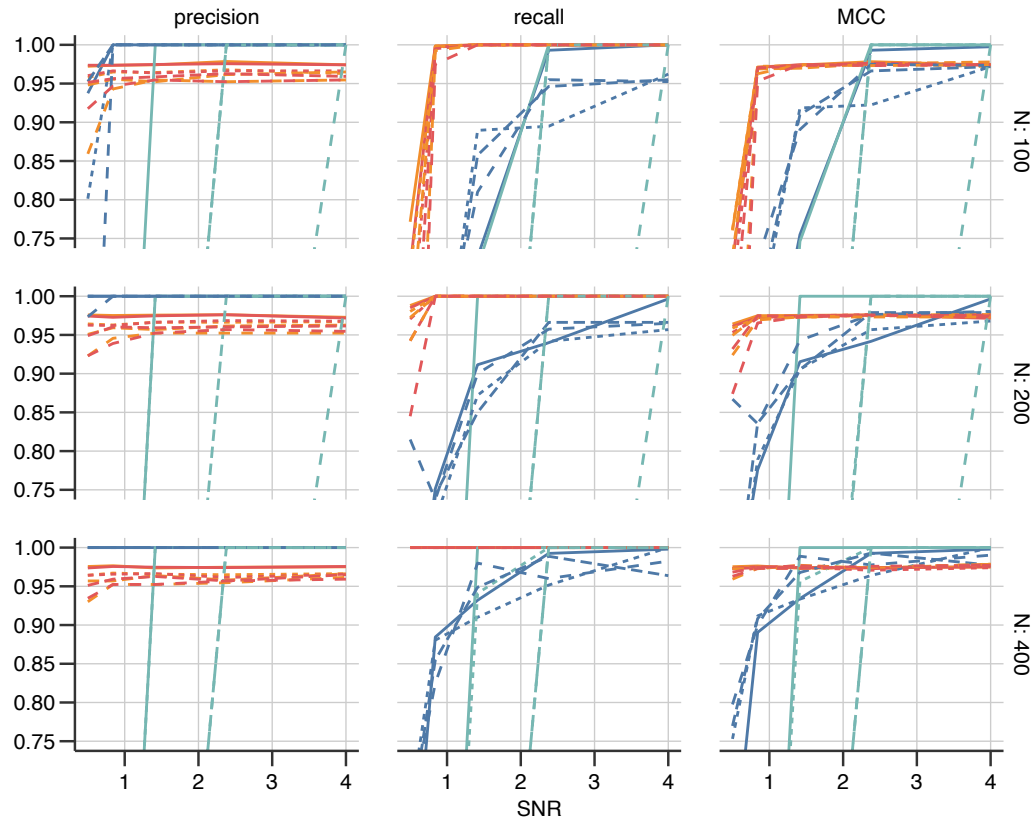

Supplement: S3 Fig — (A) Temporal ordering performance of COFE compared to three other methods (CYCLOPS [15], PAGA [24] and scPrisma[21]) measured using median absolute position error (MAPE) on data with p = 2000 features and different signal-to-noise ratios (SNR), number of samples (N) and fraction of rhythmic features (r). (B) Rhythmic feature detection performance of COFE compared to three other methods quantified by precision, recall and Matthews Correlation Coefficient (MCC), which combines precision and recall, for the same synthetic data in (A). The data underlying Figure panels can be found in S1 Data. (PDF) [file pbio.3003196.s015.pdf]

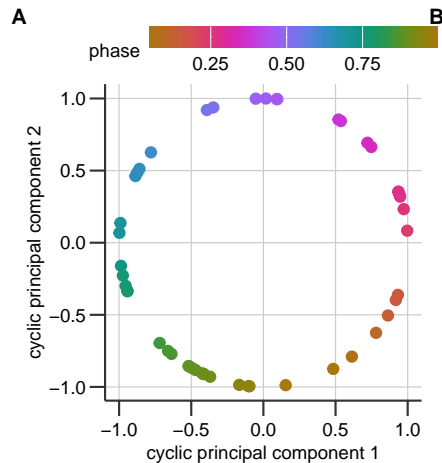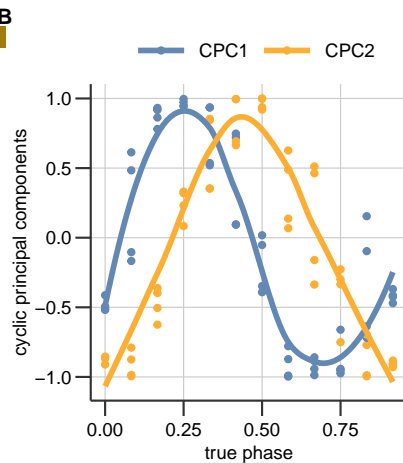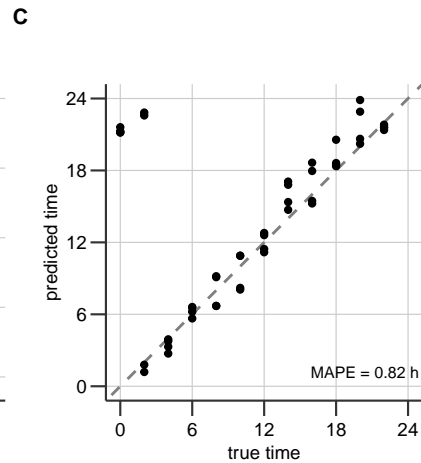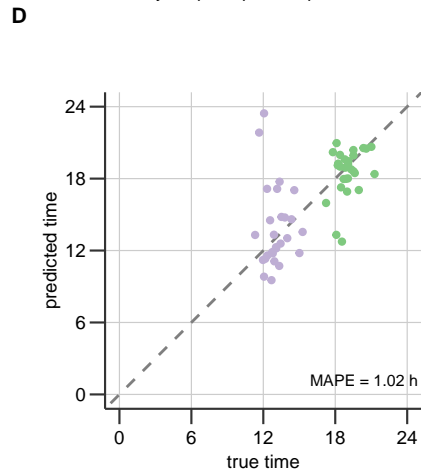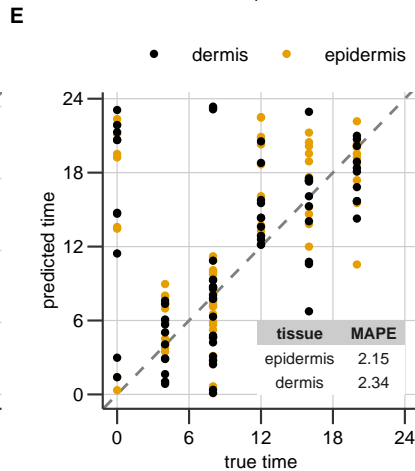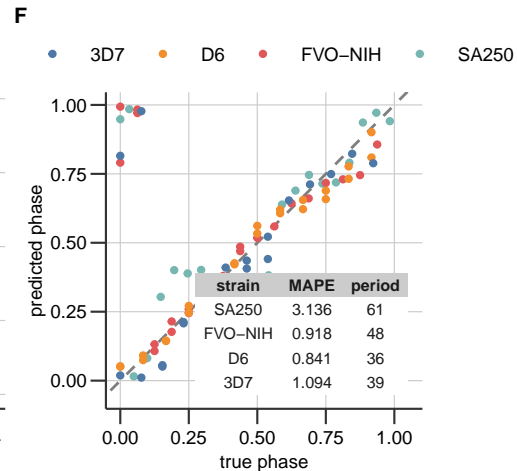

Supplement: S4 Fig — (A–C) COFE applied to the mouse liver RNA-seq data (SRP197108) collected hourly over 48h [53]. (A) The cyclic principal components (CPCs) extracted by COFE with the samples color-coded by phase within a cycle (normalized to 1.0). (B) The CPCs plotted against the true sample phase within a cycle (normalized to 1.0). (C) Scatter-plot of the true and predicted sample times. (D, E) Performance of COFE on human data. (D) COFE trained on Nanostring gene expression data from human blood monocytes is used to predict sample times for independent validation data [23]. (E) COFE applied to longitudinal time series human microarray gene expression data (GSE205155) from two different skin layers [13]. (F) Time label reconstruction of RNA-seq time-series of in-vitro cultures of four strains of P. falciparum (malaria parasite) [26]. The data underlying Figure panels can be found in S1 Data. (PDF) [file pbio.3003196.s016.pdf]

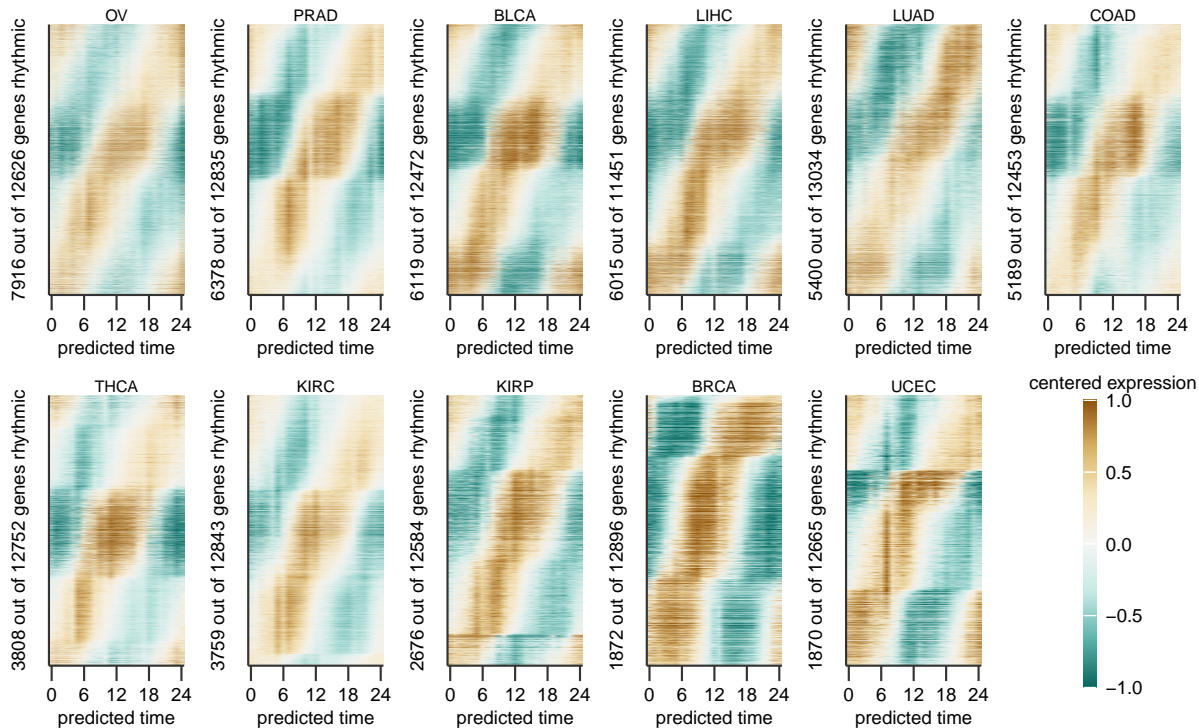

Supplement: S5 Fig — Heatmaps of gene expression patterns of rhythmic genes, which are sorted by peak time of expression in each AC, for different ACs. (PDF) [file pbio.3003196.s017.pdf]

**A**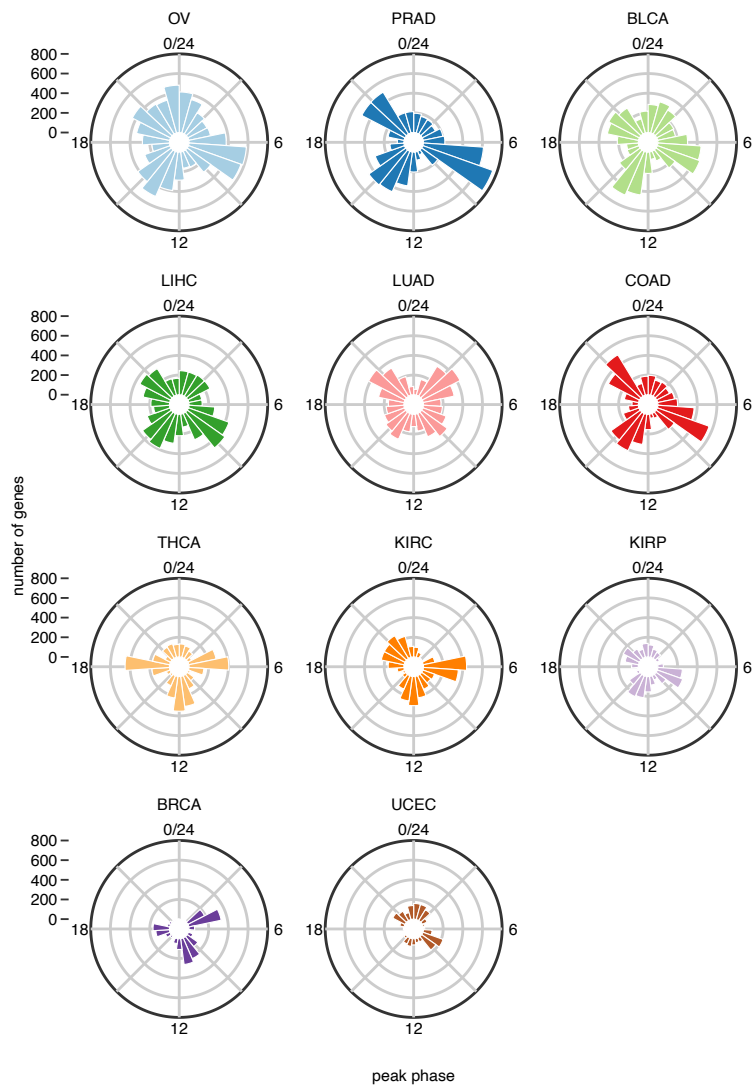**B**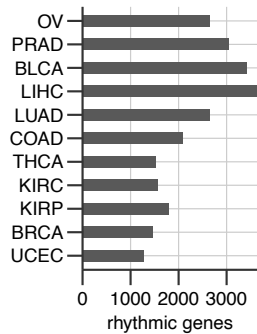**C**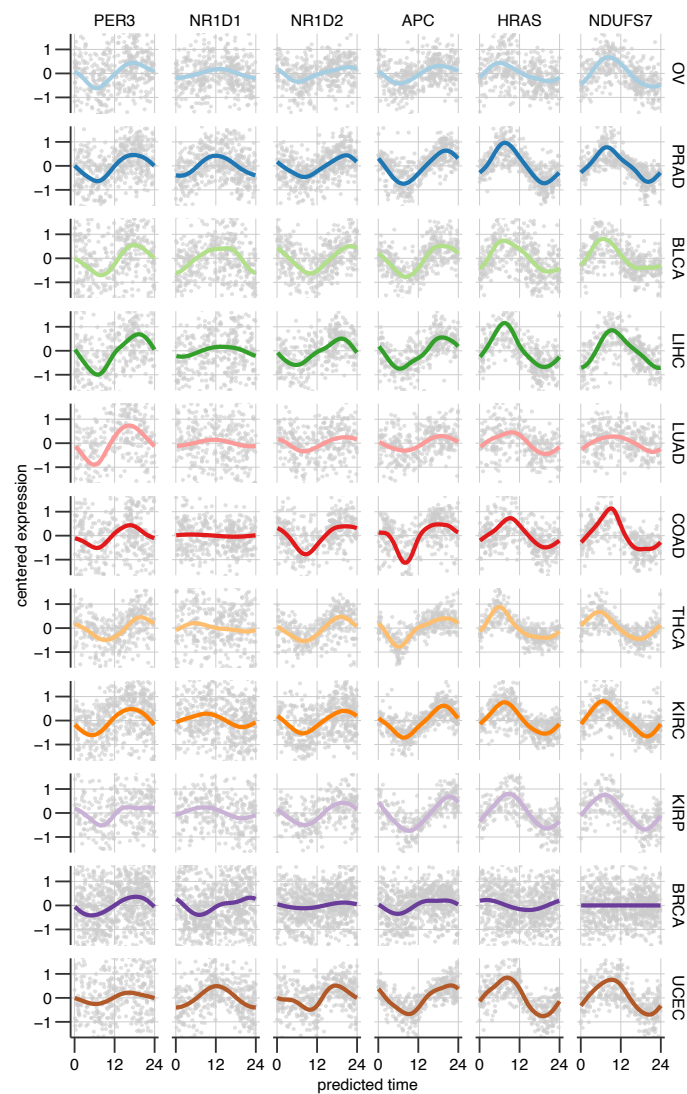**D**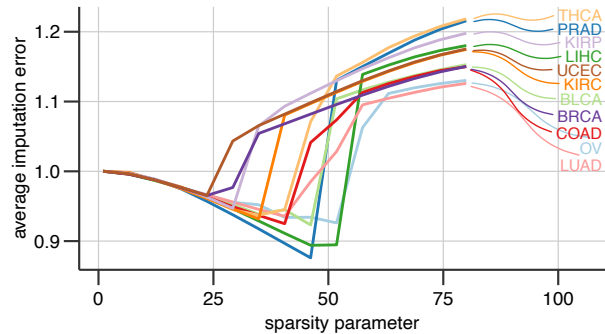

Supplement: S6 Fig — (A) Distribution of peak phase of population rhythmic genes in different ACs. (B) The number of rhythmic genes with at least two-fold peak-to-trough amplitude in the ACs (compare with Fig 1C). (C) Raw data ordered using predicted time labels with the LOESS-smoothed estimates of the mean profile for selected genes. (D) Output of the repeated 5-fold cross-validation for each AC. The data underlying Figure panels can be found in S1 Data. (PDF) [file pbio.3003196.s018.pdf]

**A**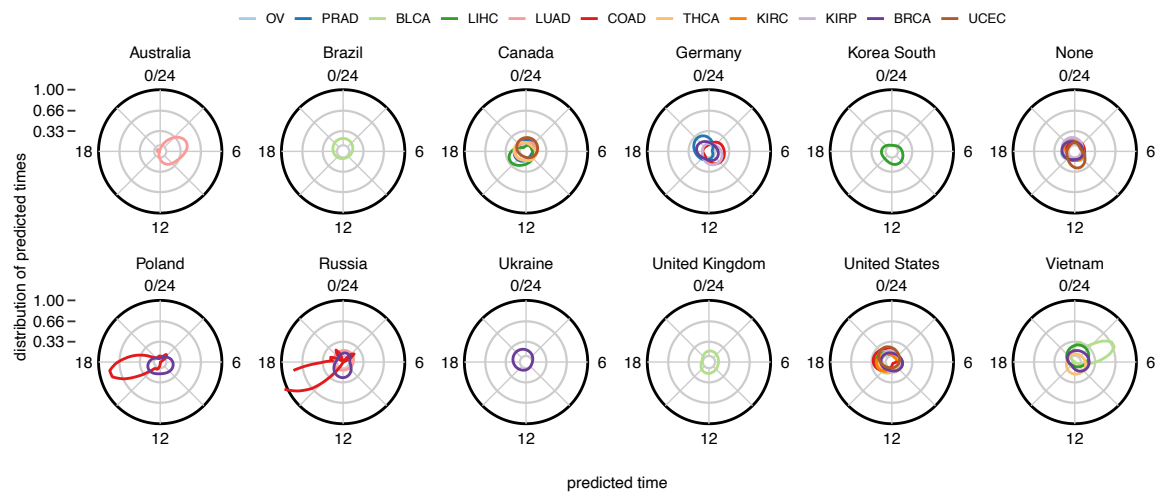**B**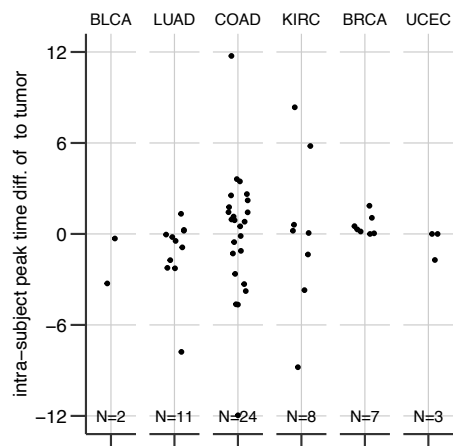**C**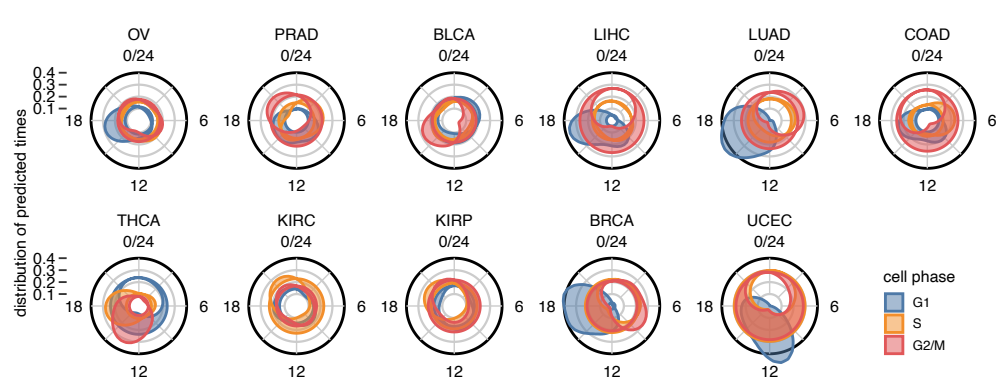**D**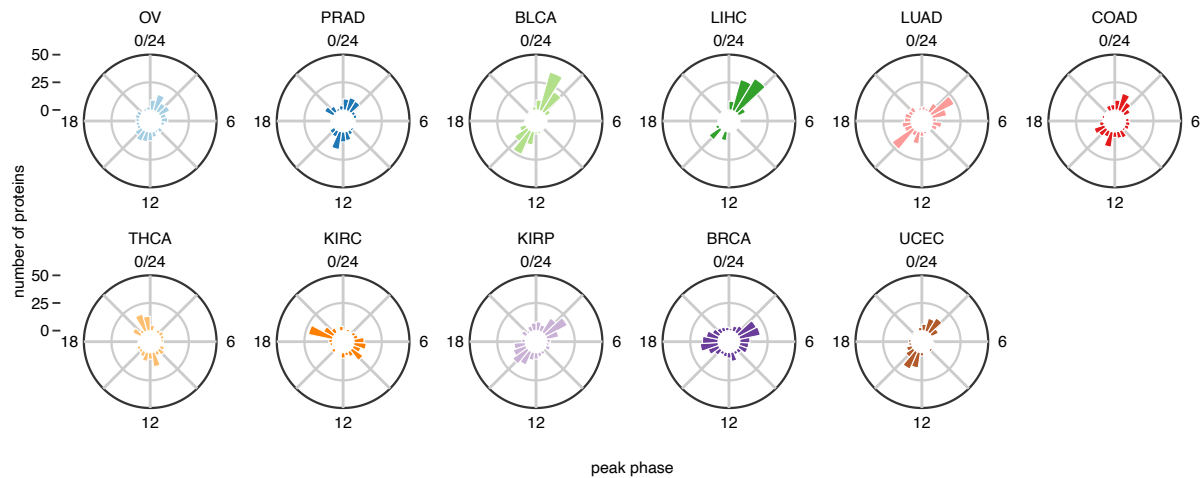

Supplement: S7 Fig — (A) Distribution of predicted time labels for the samples in each AC according to source country of the sample. (B) Phase difference predicted by COFE between cancer and patient-matched cancer samples not used for training COFE. (C) Predicted predominant cell cycle phases in the different patient samples predicted by the gene expression-based cell cycle phase scores. (D) Circular histograms of the peak time of expression of rhythmic proteins. The data underlying Figure panels can be found in S1 Data. (PDF) [file pbio.3003196.s019.pdf]

A

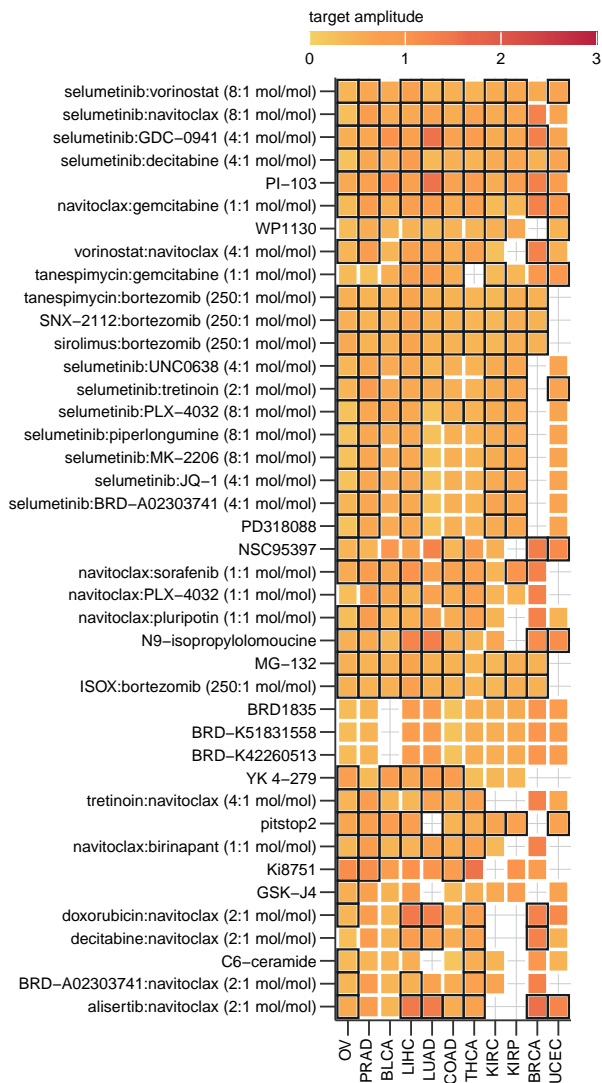

B

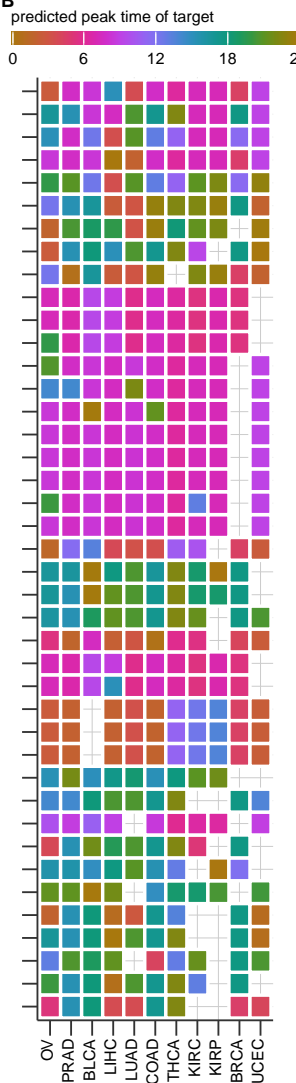

Supplement: S8 Fig — The log2-fold amplitude (A) and peak time relative to the core clock (B) of the largest rhythmic target gene in each AC of putative drugs that target cancer pathways or processes. All putative drugs with rhythmic targets in at least 9 ACs are included. Drugs with multiple rhythmic gene targets in an AC are boxed in black. The data underlying Figure panels can be found in S1 Data. (PDF) [file pbio.3003196.s020.pdf]
