# Supplementary material for: Rhythm profiling using COFE reveals multi-omic circadian rhythms in human cancers in vivo
Source: PLoS Biol. 2025 May 27;23(5):e3003196. doi: 10.1371/journal.pbio.3003196 (PMC12136439; doi:10.1371/journal.pbio.3003196)
Supplement: Attachment — Submitted filename: PLoS_Bio_Response_to_Reviewers.pdf [file pbio.3003196.s021.pdf]

Response to Reviewers - PBIOLOGY-D-24-01923R1

Bharath Ananthasubramaniam and Ramji Venkataramanan

December 17, 2024

We thank the reviewers and the editor for their constructive input and we believe that the manuscript is much improved as a result of the changes that were suggested.

Response to Editor

As you will see, the reviewers find the method novel and interesting, but they also raise several issues that would need to be addressed before we can consider the manuscript for publication.

Reviewers 1 and 2 would like to see comparisons with additional methods published in terms of novelty and advance

We have expanded on the evaluation of COFE first on synthetic data in S1 Fig., S2 Fig and S3 Fig. In S1 Fig., we have outlined the basic workings of COFE with an example and characterized its temporal ordering and rhythmic feature identification performance for different data configurations. We also quantified the negligible effect of the approximations we made in the formulation of COFE. In S2 Fig., we showcased how the cross-validation (CV) curve serves a second purpose to evaluate the quality of the ordering and flag invalid reconstructions that might arise in arrhythmic data or when data are only collected across a fraction of a cycle.

We also expanded the comparison of COFE to other methods to include a spectral analysis (scPrisma) and graph-based method (PAGA) (as suggested by reviewer 2) in S3 Fig. We emphasize how COFE is the only method that can simultaneously order the sample temporally and identify rhythmic features. In fact, COFE identifies rhythmic features with perfect precision (no false positives) over a wider range of parameters than other methods. The other methods, which order first and then identify rhythmic features, are limited by the Type I error threshold (α) chosen and must admit a few false positives. Moreover, none of the methods possess this (CV) procedure that can flag invalid temporal reconstructions.

Reviewer 1 also thinks that the claims should be experimentally validated and confirm that the method makes valid predictions

The main premise of our work is that sampling internal tissue from humans for time series rhythm analysis is clinically and ethically infeasible. In other words, barring technological advances that allow non-invasive time series sampling of internal tissue, direct experimental evidence for circadian rhythms in tumors cannot be collected. We have to compromise to either study population rhythms (in vivo), as we do, or settle for time series cell-line studies (in vitro) or time series sampling of cultures from single biopsies (ex vivo). Neither of the two latter approaches provide satisfactory and appropriate comparisons to validate our predictions, as they lack the systemic circadian context present in in vivo studies.

We have nevertheless outlined multiple lines of evidence in the Discussion that support the validity of COFE's predictions regarding circadian rhythms in the different adenocarcinomas (ACs):

- We have validated COFE on various scenarios and have confirmed that it produces accurate reconstructions, while also flagging invalid orderings (S1 Fig., S2 Fig.). The CV of COFE runs on TCGA data shows a clear minimum (S6D Fig.), which signals a valid reconstruction.
- Most core clock genes are identified as rhythmic de novo, and peak in a conserved order around the circadian cycle based on the response elements in their promoters.

- There are large statistically significant overlaps in the rhythmic clock output genes discovered independently in the 11 different adenocarcinomas by COFE.
- Our list of high amplitude rhythmic clock genes in BRCA overlapped significantly with those in a recent study [1].
- The protein products of the identified rhythmic genes were also highly likely to be rhythmic and they were consistent in their peak time of expression. This association of de novo rhythms in the transcriptome and cancer-specific proteome further supports our conclusions.

Reviewer 2 asks for further details on the method to allow readers to reproduce it, and suggests some improvements that would be important to clarify the results.

We have made a number of changes to elaborate on the method. We have included a detailed schematic of the method as a new Fig. 1. We have also expanded the Theory (Pages 13-14) and the Implementation (Pages 15-16) parts of the Materials and Methods section to make the derivation of the method clearer. To further support the mathematical grounding of the Theory section, we have provided a new supplementary text (S1 Text). Finally, we have provided pseudocode for the scPCA algorithm to further help with reproduction of the method (S2 Text) and a Jupyter notebook "Getting started with COFE on synthetic data" on the COFE repository on GitHub to facilitate understanding of the method and use of the Python package.

Reviewer 3 thinks you should explore the performance of COFE as a function of the observed fraction of cycle in simulated data, along with discussing the implications for the TCGA application and clarifying several points.

This insightful comment led us to further explore our cross-validation (CV) approach. This revealed that CV is able to flag invalid orderings of the kind the reviewer identified (datasets with no rhythmic features or sampling over only a fraction of the cycle). We have showed in on synthetic data how the CV is able to do this (S2D-H Fig.). For the TCGA data, we verified that the CV indeed indicates a successful ordering (S6D Fig.) suggesting that data appear to be collected around the circadian cycle. We propose in the Discussion that circadian rhythms in the ACs are decoupled from the healthy tissue clocks, resulting in AC internal clock times apparently spread throughout the circadian cycle.

Response to Reviewers

Reviewer 1

This manuscript presents a method, COFE, for time-series free circadian analysis particularly of omics data. The method relies on determining two principal components, and in that way it is similar in spirit to other methods. The authors then apply the method to existing databases, such as the TCGA or Reactome database. From this, they make claims about cyclic components within these databases and, for example, the relationship between cycling in the cell cycle and circadian clock.

I struggled quite a bit with this manuscript. While on the surface, there are claims that might be of broad interest to the readership of PLOS Biology, few in the time series or signal processing communities would accept their method as is. Indeed, there are large communities devoted to this kind of work and questions of inferring rhythms from limited data, the SAMPTA conference might be the best, and there are many mathematical concerns that are not addressed such as topological considerations, questions about the shape of rhythms and harmonics, and the relationship to other methods. There is some benchmarking against other methods. It is not always completely clear that this is a better method, and there are many additional methods, even especially for circadian analysis, to compare to. The study of synthetic data needs to be significantly expanded to be confident in the method. I'd be less concerned about this if the manuscript was focused on a single biological question and there was other new data to support the predictions of the method.

No new data is presented, and even if the method were more mathematically validated as others are, I think PLOS Biology readers would want some further experimental validation of their claims. One

promising feature is that the circadian genes have phases similar to those found in other studies, but this is not enough for the very broad claims that are made in Figure 3 about candidate cancer drugs.

In summary, a lot more work needs to be done before it can be a convincing method broadly used. I encourage the authors to work towards this. I suggest breaking off the parts of the manuscript, focusing on the method, and getting validation by publishing them in the time series literature or statistics literature as a general method. I would then consider a manuscript where more biological details can be explored (perhaps in a full manuscript format), for example, through new data or strong supporting evidence from other studies. With that, readers would see that the method makes validated predictions, other readers would be interested in using the methods in other contexts, and such a manuscript might find a welcome home in PLOS Biology or a similar journal.

We agree that inferring rhythms from limited data has been studied widely, but the signal processing and time series literature on this problem primarily considers *time labeled* data. For the setting where time labels are available, a variety of approaches have been proposed (and applied to biological time series), including parametric tests [2], permutation tests [3], non-parametric tests [4], tests based on spectral estimators [5, 6], and topological approaches based on ordering or persistent homology [7]. A comparison of a few popular algorithms for detecting rhythms in time labeled biological data can be found in [8]. We also perused the proceedings of SAMPTA from 2013 onwards, and were unable to find methods that can be applied for inferring rhythms in data without time labels.

In contrast to the above methods, COFE is unsupervised and infers time labels and learns rhythmic features from data *without* time labels. We have now fixed potentially misleading text in the Introduction and replaced ‘reordering’ with ‘ordering’ to describe COFE’s functionality throughout the text. The methods designed for data without time labels (circular-autoencoder based CYCLOPS [9], expectation-maximization-based CHIRAL [10] and circular-topology-based CIRCLUST [11]) all require a priori assumptions regarding the identity of the rhythmic features. On the other hand, most analogous algorithms in the single-cell RNA-seq literature are primarily concerned with sequentially ordering samples (cells) in time rather than assigning time labels per se [12–15]. COFE is able to reconstruct rhythms and find rhythmic features de novo, which has also been shown to be statistically superior [16] to assigning time labels first and then applying one of the methods for data with time labels mentioned above.

The main premise of our work is that sampling internal tissue from humans for time series rhythm analysis is clinically and ethically infeasible. In other words, barring technological advances that allow non-invasive time series sampling of internal tissue, direct experimental evidence for circadian rhythms in tumors cannot be collected. We have to compromise to either study population rhythm (in vivo), as we do, or settle for time series cell-line studies (in vitro) or time series sampling of cultures from single biopsies (ex vivo). Neither of the two latter approaches provide satisfactory and appropriate comparisons to validate our predictions, as they lack the systemic circadian context present in in vivo. Nonetheless, we have outlined multiple lines of evidence in the Discussion that support our conclusions regarding circadian rhythms in the different adenocarcinomas (ACs):

- We have validated COFE on various scenarios and have confirmed that it produces accurate reconstructions, while also flagging invalid orderings (S1 Fig., S2 Fig.). The CV of COFE runs on TCGA data shows a clear minimum (S6D Fig.), which signals a valid reconstruction.
- Most of the core clock genes are identified to be rhythmic de novo and peak in a conserved order around the circadian cycle based on the response elements in their promoters.
- There are large statistically significant overlaps in the rhythmic clock output genes discovered independently in the 11 different adenocarcinomas by COFE.
- Our list of high amplitude rhythmic clock genes in BRCA overlapped significantly with those in a recent study [1].
- The protein products of the identified rhythmic genes were also highly likely to be rhythmic and they were consistent in their time of peak expression. This association of de novo rhythms in the transcriptome and cancer-specific proteome further supports our conclusions.

In our results, we also highlighted, which cancer drugs and their associated cancers might benefit from considering time of administration in the overall treatment. However, preclinical and clinical trials would be necessary to successfully screen and find drugs suited to such a chronotherapy paradigm, which is beyond the scope of our study and expertise.

We have now expanded on the Theory part of the Materials and Methods section and added a supplementary section (S1 Text) to elaborate on the mathematical foundation of COFE. In addition, we have also expanded on the synthetic data validation and comparison to other methods (S1 Fig., S2 Fig., S3 Fig.). As indicated earlier, there has been recent work on detecting or reconstructing rhythms from data without time labels, primarily in a biological context, e.g., Cyclops, Oscope, Cyclum, Paga, and scPrisma [9, 12–15]. None of these methods too have been mathematically validated via a theoretical analysis. We have included most of these in our synthetic data validation of COFE (S3 Fig.). We agree that a mathematical analysis of COFE is an important direction for future work, but would like to highlight a few points that make COFE already statistically well-grounded and more interpretable than methods based on neural networks, e.g., Cyclops and Cyclum.

- The sparse cyclic PCA (scPCA) used by COFE is a natural generalization of the widely used sparse PCA, obtained by adding the entry-wise cyclic constraint for the two principal components. Similarly, the alternating maximization algorithm we use for the biconvex optimization underlying scPCA is a natural generalization of the SCoTLASS algorithm for sparse PCA [17, 18].

- We have now detailed in S1 Text that scPCA can be viewed as a way to estimate parameters in the parametric cosinor regression model [2] when the features (time labels) are unknown Eq. (S1). Cosinor regression is commonly used in circadian analysis for identifying rhythmic features when the time labels are available, with the model in Eq. (S1) interpreted as a Fourier series approximation of a periodic signal at the fundamental frequency.

- In the revised manuscript, we discuss the cross-validation (CV) procedure in more depth and show how it quantifies the quality of the ordering. As S2 Fig. illustrates, a sharp minimum in the CV error curve indicates good re-ordering, while a relatively flat CV curve suggests that the ordering is potentially invalid, such as a dataset with no rhythmic features or sampling of only a fraction of the cycle. None of the other methods have a cross-validation procedure that can flag invalid ordering.

- COFE is the only method that can simultaneously order the sample temporally and identify rhythmic features. In fact, COFE identifies rhythmic features with perfect precision (no false positives) over a wider range of parameters than other methods. The other methods rely on ordering first and then identify rhythmic features are limited by the Type I error threshold (α) chosen and must admit a few false positives.

Reviewer 2

Single-cell RNA sequencing (scRNA-seq) data typically provides a snapshot of cellular states without temporal information. However, given its massive amount of information, recent efforts have focused on extracting temporal dynamics, such as circadian rhythms, from scRNA-seq data. The author introduced COFE (Cyclic Ordering with Feature Extraction), which orders cells according to their circadian phase while simultaneously identifying rhythmic features. Applying COFE to 11 different human adenocarcinomas (ACs) identified 2,000 to 6,000 rhythmic genes in each AC. The amplitude and peak time of these rhythmic genes were further analyzed using Gene Set Enrichment Analysis (GSEA) with the Reactome database, leading to the identification of enriched pathways associated with metabolism, the cell cycle, and the immune system. The results are very interesting and the developed method is very useful. However, the explanation for the method seems not sufficient to understand the algorithm fully and reproduce the results.

To address this issue, we have significantly expanded on the Materials and Methods sections dealing with the Theory and Implementation. We have added a more thorough schematic of the method in a new Fig. 1, a computational example in the form of a Jupyter notebook in COFE GitHub repository and pseudocode of the key methods that perform sparse cyclic PCA to further help the reader. We give the details of these changes in the responses to specific comments.

Major

1. The author developed COFE for pseudo-time inference in scRNA-seq data. Although a benchmark study was performed comparing this algorithm with CYCLOPS, there are numerous other popular algorithms for pseudo-time inference, ranging from conventional trajectory analysis methods such as PAGA [15] to newer approaches such as scPrisma [14] Therefore, performing an additional benchmarking study that includes widely used tools, such as PAGA and scPrisma, would help to better demonstrate the novelty of COFE.

   We first want to clarify that while COFE indeed is applicable to all high-throughput data sources including scRNA-seq data, the current method and software implementation do not account for specific features of scRNA-seq data, such as missing values/zero-inflation. Hence, we currently recommend that the method be used only for data that satisfy our additive noise model in S1 Text.

   We note that our COFE implementation can be easily extended to handle missing values. Indeed, our cross validation procedure uses the imputation error for (artifically) deleted values to optimize the sparsity hyperparameter. The same procedure could be used to impute missing values in the data, but we leave this extension for future work.

   We have now expanded our benchmarking study to include both PAGA and scPrisma and present it in S3 Fig.. We did not include Cyclum [12] in our benchmarking due to its remarkable similarity to CYCLOPS (both are circular autoencoder with slight differences). In summary, we found that PAGA and CYCLOPS performed very similarly, with very good recall performance but with precision limited by the Type I errors of the statistical cosinor test for rhythmicity (i.e, the chosen α). COFE and scPrisma provide perfect precision over a range of SNRs, number of samples and fraction of rhythmic features, but imperfect recall. Moreover, scPrisma's ordering performance of samples was generally poor (as the method itself is not designed to assign phase estimates to samples) and tended to drop rapidly at low SNR, low rhythmic features and low sample sizes. We have elaborated on the benchmarking now in the main text (Page 3-4).

2. page 12 - 3rd paragraph (2nd line) In "Theory" section of the Materials and Methods: The authors ignored the term related to the orthogonality of singular vectors in equation (1) simply because they are difficult to deal with. This omission could compromise optimal data compression, as well as amplify periodic signals by exaggerating certain principal components (PCs). Therefore, please explain in more detail the reason for the omission of these terms or cite the paper if possible.

   We now discuss the effect of our omission of the cross term in a new "Relationship between the simplified optimization and the matrix approximation" subsection (Page 15). In summary:

   (a) As has been observed previously with sparse PCA [18], one part of the cross term, $v_1^T v_2$, is typically close to zero, due to the sparsity enforced on the two loading vectors independently. In other words, orthogonality of the principal components is obtained practically for free. Moreover, explicitly enforcing orthogonality often leads to less sparse solutions, which is also not desirable.

   (b) In practice, the value of the cross-term for the solution found by COFE constitutes less than 0.1% of the matrix approximation error in synthetic data simulations (S1I Fig.) and in biological cancer data from TCGA (S6 Table).

(c) We minimize the effect of this approximation further by choosing our best solution from multiple *restarts* as the one with the best matrix approximation error, rather than the simplified score used to find a fast procedure (Page 15-16).

We therefore conclude that the simple, fast scPCA procedure finds excellent solutions for the general problem in Equations (1) and (2).

3. page 13 - 1st paragraph (1st line) In ”Alternating maximization” section of the Materials and Methods: There is no explanation of which distribution was used to generate the initial conditions for u and v. Furthermore, the following steps are difficult to understand based on the current description, particularly regarding the algorithm used for maximizing equation (2). For reproducibility, it would be helpful to provide a detailed explanation of how the u and v vectors are initialized and updated, as well as which algorithm was used for the maximization, possibly including pseudocode or a flow chart.

   To make the steps in COFE clearer, as the reviewer requested, we have separated the old Fig. 1A into a new Fig. 1. We have also included the pseudocode of the sparse cyclic PCA algorithm in the S2 Text. In the “Implementation — COFE” section of Materials & Methods, we describe in greater detail how we implemented scPCA.

   To the reviewer’s specific question: we initialized the elements of $\mathbf{v}$s with random draws from a sparsity-encouraging Laplacian distribution that are subsequently scaled to satisfy the constraints, and we chose the pairs $(\mathbf{u}_{1i}, \mathbf{u}_{2i})$ randomly to lie on the unit circle.

   We have elaborated in the Alternate Maximization section of the Materials & Methods (Page 14) how we mathematically solve the equations using Lagrange multipliers and an algorithm described in Guillemot et al. rather than perform a brute-force optimization.

4. page 13 - 1st paragraph (1st line) In “Alternating maximization with regularization” section: If v is not a binary vector, it seems too arbitrary to select the gene corresponding to a non-zero element of v as a rhythmic gene. For instance, if an element of v is close to zero but not exactly zero (e.g., 0.0000001), can it truly be considered a rhythmic gene?

   We understand the spirit of the reviewer’s question and we too have wondered about doing this. However, we do not favor this approach for two reasons.

   (a) The sparsity constraint (l_1 constraint on $\mathbf{v}$s) already thresholds and sets many coefficients to zero. One might set an additional threshold, as the reviewer suggests, but its choice would be rather arbitrary. Moreover, enforcing sparsity using the l_1 constraint, without additional thresholding, is consistent with applications of standard sparse PCA (without the circular constraint) [18].

   (b) The cross-validation curve (see for e.g., S1B Fig.) shows that setting more elements of $\mathbf{v}_1, \mathbf{v}_2$ to zero (i.e., using a smaller s) leads to larger cross-validation error making such a change suboptimal in our formulation; recall we chose the optimal s based on the minimum of the cross-validation error.

5. page 14 - 1st line in (iv) of “Implementation - Cyclic Ordering with Feature Extraction (COFE)” section: The author introduces s as the sparsity level. However, as s decreases, the l1 norm of vector v also decreases, which, according to equation (6), indicates a higher level of sparsity. To clarify this, it would be beneficial to elaborate on the relationship between the parameter s and the level of data sparsity in the manuscript.

   In the new schematic of the method in Fig. 1, we indicate the inverse relationship between the sparsity (hyper)parameter s and the level of sparsity.

   As we describe in the Materials and Methods, we would like to enforce a limit on the number of features (i.e., l_0 constraint on the $\mathbf{v}$s) but that is intractable. So, we followed the standard practice

of replacing it with limit on the sum of the absolute weights (i.e., l_1 constraint on the $\mathbf{v}$s). A consequence of this change is that the strict relationship between number of included features (sparsity level) and sparsity parameter s is lost.

Nevertheless, we can perform some back-of-the-envelope calculations. Recall that we impose both $\|\mathbf{v}\|_1 = s$ and $\|\mathbf{v}\|_2 = 1$. For the highly simplified situation where the M included features all have the same absolute weight (i.e, $= \pm m$),

$$\|\mathbf{v}\|_2 = 1 \implies \sum m^2 = 1 \implies m = \frac{1}{\sqrt{M}}.$$

Then $\|\mathbf{v}\|_1 = M/\sqrt{M} = \sqrt{M} = s$. Thus, the number of included features M scales approximately as the square of the sparsity parameter s, which bears out on runs of COFE on the synthetic data under different data configurations (S2C Fig.). We have included this discussion in the results section under the validation of COFE (Page 3, S2B,C Fig.).

6. page 14 - 3rd line in (iv) of "Implementation—Cyclic Ordering with Feature Extraction (COFE)" section: There is no description of how random elements were deleted from the data for cross-validation (CV). How were these elements selected and deleted? Were elements deleted regardless of their value, or did the probability of deletion vary based on the magnitude of the element's value? Additionally, is there a relationship between the parameter s and the number of deleted elements?

   We have expanded on the "Implementation — COFE" section (Pg. 15) to explain the CV procedure in greater detail. The number of deleted elements is always $1/K$ for K-fold CV and are deleted independent of s or the value of the element. Elements are chosen for deletion such that they are evenly spread across the rows (samples) and columns (features). This is achieved by circular shifts of pseudo-diagonals of the data matrix defined in [19].

7. page 3 and 4 - in Widespread in vivo population rhythms in human adenocarcinomas rhythmic gene section: After applying COFE, the author extracted the amplitudes and peak times from genes ordered by their pseudo-time and used these quantities for analysis. However, there is no explanation of how the amplitude and peak time of each gene were determined. As these quantities are used extensively in this study, it is essential to describe the steps for obtaining amplitude and peak time from the ordered genes.

   We have elaborated on the steps to estimate circadian parameters in greater detail in section D of S4 Text. Samples in the data are assigned time-labels by COFE, which results in time-series for all the features of interest. We fit a cosine function to the expression time series of each feature to extract circadian parameters.

Minor

1. page 4: Figure 1A does not clearly explain the method. A dedicated figure illustrating the different steps of the algorithm would likely be more effective. For example, the v vectors were incorrectly described as binary, but they are not in Fig. 1a. Additionally, the description of the gray panel used to determine the sparsity parameter in Fig. 1A is missing from both the caption and the result section, making it difficult to interpret the role of this step in the COFE. It would be better to add this description to the figure caption.

   We have created a dedicated Fig. 1 to explain COFE as suggested by the reviewer and fixed the highlighted issues.

2. page 4: The vertical colored line in Figure 1F makes it difficult to determine which data has been selected.

   We have removed the vertical lines.

3. page 3 - 1st paragraph (4th line) in “Widespread in vivo population rhythms in human adenocarcinomas” section: “which are cancers of epithelial origin that develop in organs and glands and account for 80 to 90 percent of all cancers ”: reference?

   This statistic was obtained from the NIH’s National Cancer Institute’s SEER training modules and has now been cited in the text.

4. page 6 - 1st paragraph (1st line) in “Key cellular processes are enriched for rhythmic genes” section: “To identify the processes enriched for rhythmic genes in different ACs, we performed gene set enrichment analysis [25] using the Reactome database”. (a) Put the database version as it is frequently updated. (b) What tools and GMT files are used? (Java application, R package, ...)

   We obtained Reactome database annotations via the reactome.db package in Bioconductor. We have now added this information in the main text and also included a list of R packages used in the analysis and their version numbers in the Supporting Information (S7 Table).

5. 13 page - 1st line in (i) of ”Implementation - Cyclic Ordering with Feature Extraction (COFE)” section: Before applying COFE, data was standardized. However, the specific standardization method used is not clearly described, even though various approaches, such as log-normalization and scTransform, are available to stabilize data variance. Furthermore, standardization can distort signals in sparse data, potentially leading to false discovery, as noted in a recent study [Kim et al., 2024]. Therefore, it is necessary to specify the method used for data standardization and to explain how any potential distortion in sparse data was addressed.

   We have now described clearly and highlighted the preprocessing steps in the schematic in Fig. 1.

   We thank the reviewer bringing this study to our attention. The current version of COFE does not account for the sparse nature of data, and therefore is recommended only for datasets that are well modeled by our additive noise model described in S1 Text. For this reason, we applied COFE only to bulk RNA-seq data from different adenocarcinomas in this study. However, adapting COFE for scRNA-seq data is an exciting direction of future work.

6. If available, put the version of recount3

   We obtained recount3 data via the bioconductor R page, which we have now cited in the text.

7. page 8 - 2 paragraph of ”Protein expression is rhythmic, even in ACs with few rhythmic genes”: The authors wrote, ”Activated forms of members of key signaling pathways, such as AKT, EGFR, and MAPK, were also rhythmic in almost all ACs.” These three pathways are well-known to be deregulated in cancer. More information about the different proteins involved in these pathways and found rhythmic could be added. The authors may want to add the name of the most important rhythmic proteins, the results they found, their involvement, and the consequences on the pathway. They can further develop by showing how this is relevant for cancer studies.

   We have now categorized the rhythmic proteins according to the classification in [20]. We found multiple proteins and activated forms from 7 different pathways that were rhythmic in 9 or more ACs, which we have listed in the text (Pages 9-10). We have also annotated all the rhythmic proteins with the ‘Pathway’ they belong to in the S2 Table. These widespread rhythms across key cancer-relevant pathways raise questions about how exactly to interpret previous observations of their misregulation in cancer. Furthermore, taking the rhythms into account could help us better stratify individuals, especially since many of these proteins are used a prognostic markers for cancer progression. We have now included these points in the Discussion.

Reviewer 3

This paper presents a novel method for reconstructing temporal information in a population of untimed high-dimensional (e.g., RNAseq) data by projecting it onto a circular manifold. By enforcing sparsity in

the projection, it is possible to recover the features that are the most strongly periodic, without the need for a second stage analysis. Applied to data from TCGA, the authors discover rhythmicity in tumor cell gene expression that differs between healthy and normal tissue; because the proteins associated with this rhythmic expression may be chemotherapy targets, this points the way toward timed dosing regimens that may be more effective with fewer side effects. All in all, I find this to be an excellent contribution to the field.

I do have a few questions on the methodological side:

1. The projection of the samples onto the circle assumes that they are collected "around the clock". What happens if they are not, that is, if only a portion of the cycle is observed? With the possible exception of postmortem samples, the vast majority of samples will tend to be collected between 9am and 5pm – only 1/3 of the cycle observed. The performance of COFE as a function of the observed fraction of cycle should be explored in simulated data, and the implications for the TCGA application should be discussed.

   This is a great point, thanks to the reviewer for the suggestion. We performed a simulation on data with samples collected for different fractions of the day (for SNR=$\sqrt{2}$, $N = 200$ samples and $r = 0.2$ fraction of rhythmic features). The results (S2E-H Fig.) illustrate how the cross-validation (CV) curve can flag such situations; the CV curve does not show a clear minimum. For the TCGA data, we verified that we do obtain a clear minimum in the CV curve (S6D Fig.).

   The samples appear to be collected around the clock, although we might reasonably expect, as the reviewer indicates, that the samples were predominantly collected during the day, albeit with differences across countries. We hypothesize that the clocks in cancer tissue are free-running with respect to the rest of the circadian system, which continues to be entrained to the day-night cycle, resulting in samples appearing to be collected throughout the day. This hypothesis is also supported by lack of fixed phase relationship between healthy and cancer clocks (Fig. 3B). We added this point to the discussion on Page 12.

   We have now emphasized this role played by the CV curve in the workflow (new Fig. 1).

2. COFE's performance was investigated in synthetic data with as little as r=0.1 fraction of cycling features. What happens if there are NO cycling features, such as in a case where (for example) the circadian rhythm is severely compromised? It will of course map the data onto a circle (because it has no choice but to do so), but then what? And can one tell if there is NOT a strong signal? (Quantification of the amount of variation explained by the two circular PCs may be useful.)

   This comment by the reviewer again allows us to highlight the unique monitoring feature offered by the CV in COFE. Similar to the previous comment, we ran COFE on simulated data with different number of samples and show how CV signals the lack of a legitimate ordering of the data without a clear minimum in the CV output (S2D Fig.). We have now discussed this aspect in the Results (Page 4) and included the plots of the CV with no rhythms (S2D Fig.).

3. How does COFE perform if there are multiple rhythms with different frequencies present in the data?

   The formulation of COFE and scPCA is based on the assumption that there is only a single rhythmic dominant frequency in the data (S1 Text). In line with this understanding, COFE performs inconsistently when presented with data with, for example, two harmonic frequencies. The output of the CV displays a clear minimum and is therefore unable to detect multiple rhythmicity. COFE applied with this "optimal sparsity level" either locks on to the harmonic, the correct fundamental or a combination of the two frequencies (Fig. R1).

   We have now included this observation as a point for future improvement for COFE in the Discussion.

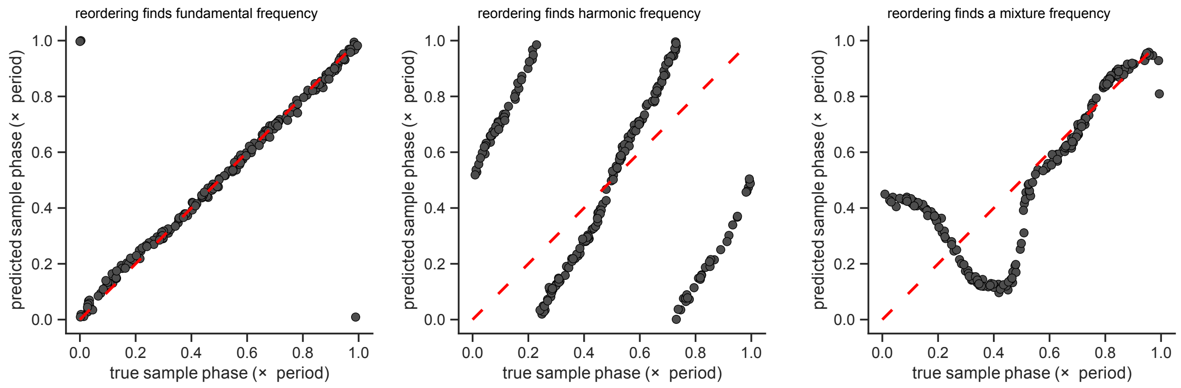

Figure R1: Different outcomes of temporal reconstruction of different synthetic datasets with two harmonic frequencies (24h and 12h). The datasets all consisted of $N = 200$ samples, $p = 2000$ features, of which 10% were rhythmic in each of the two frequencies, and SNR=1.4.

4. The optimization finds only local minima, and thus depends on the initialization; the authors report that they perform multiple restarts and "pick the solution with the best score," which I assume means the one with the greatest maximum of the objective function. How many restarts, and how rugged is the landscape?

   We used 5 restarts as the default in COFE – the number of restarts is a user-defined parameter in the software. In our experience, 5 restarts appear to provide stable, reproducible results.

   The high-dimensional nature of the landscape cannot be easily visualized. So, we show in Table R1 below the standard deviation of the matrix approximation error over 5 restarts for different values of the sparsity parameter for an example dataset with $N = 200$ samples and $p = 2000$ features, of which 20% are rhythmic. This data demonstrates that our local optimization algorithm reliably finds a good solution in a potentially fairly "smooth" landscape.

sparsity parameter	mean (matrix approx. error)	st dev.(matrix approx. error)	coefficient of variation
1.00	399680.49	3.15	7.88e-06
2.53	398551.92	208.31	5.23e-04
4.05	396322.55	5.27	1.33e-05
5.58	393351.77	11.50	2.92e-05
7.11	389645.69	22.08	5.67e-05
8.63	385273.06	10.62	2.76e-05
10.16	380365.22	11.49	3.02e-05
11.68	375088.87	0.052	1.38e-07
13.21	369937.33	2.38	6.43e-06
14.74	366493.89	18.73	5.11e-05
16.26	366055.91	25.43	6.95e-05
17.79	366624.61	23.68	6.46e-05
19.32	366488.94	29.60	8.08e-05
20.84	366304.47	20.40	5.57e-05
22.37	366206.27	14.05	3.84e-05
23.89	366181.62	8.12	2.22e-05
25.42	366211.28	4.13	1.13e-05
26.95	366254.50	34.94	9.54e-05
28.47	366254.58	5.51	1.50e-05
30.00	366244.65	23.18	6.33e-05

Table R1: Variability in the matrix approximation error across 5 restarts of the scPCA algorithm on a sample synthetic dataset for different values of the sparsity parameter.

References

1. Li, S.-Y., Hammarlund, J. A., Wu, G., Lian, J.-W., Howell, S. J., Clarke, R. B., Adamson, A. D., Gonçalves, C. F., Hogenesch, J. B., Anafi, R. C. & Meng, Q.-J. Tumor circadian clock strength influences metastatic potential and predicts patient prognosis in luminal A breast cancer. en. *Proceedings of the National Academy of Sciences* **121,** e2311854121. ISSN: 0027-8424, 1091-6490. (2024) (Feb. 2024).
2. Cornelissen, G. Applications of cosinor rhythmometry in pharmacology. en. *Journal of Pharmacokinetics and Pharmacodynamics* **48,** 339–359. ISSN: 1567-567X, 1573-8744. (2021) (June 2021).
3. Ptitsyn, A. A., Zvonic, S. & Gimble, J. M. Permutation test for periodicity in short time series data. en. *BMC Bioinformatics* **7,** S10. ISSN: 1471-2105. (2024) (Sept. 2006).
4. Thaben, P. F. & Westermark, P. O. Detecting Rhythms in Time Series with RAIN. en. *Journal of Biological Rhythms,* 0748730414553029. ISSN: 0748-7304, 1552-4531. (2014) (Oct. 2014).
5. Wichert, S., Fokianos, K. & Strimmer, K. Identifying periodically expressed transcripts in microarray time series data. en. *Bioinformatics* **20,** 5–20. ISSN: 1367-4811, 1367-4803. (2024) (Jan. 2004).
6. Ahdesmäki, M., Lähdesmäki, H., Pearson, R., Huttunen, H. & Yli-Harja, O. Robust detection of periodic time series measured from biological systems. en. *BMC Bioinformatics* **6,** 117. ISSN: 1471-2105. (2024) (May 2005).
7. Cohen-Steiner, D., Edelsbrunner, H., Harer, J. & Mileyko, Y. Lipschitz Functions Have L p -Stable Persistence. en. *Foundations of Computational Mathematics* **10,** 127–139. ISSN: 1615-3375, 1615-3383. (2024) (Apr. 2010).

8. Deckard, A., Anafi, R. C., Hogenesch, J. B., Haase, S. B. & Harer, J. Design and analysis of large-scale biological rhythm studies: a comparison of algorithms for detecting periodic signals in biological data. en. *Bioinformatics* **29,** 3174–3180. ISSN: 1367-4811, 1367-4803. (2024) (Dec. 2013).

9. Anafi, R. C., Francey, L. J., Hogenesch, J. B. & Kim, J. CYCLOPS reveals human transcriptional rhythms in health and disease. en. *Proceedings of the National Academy of Sciences* **114,** 5312–5317. ISSN: 0027-8424, 1091-6490. (2017) (May 2017).

10. Talamanca, L., Gobet, C. & Naef, F. Sex-dimorphic and age-dependent organization of 24-hour gene expression rhythms in humans. en. *Science* **379,** 478–483. ISSN: 0036-8075, 1095-9203. (2023) (Feb. 2023).

11. Larriba, Y., Mason, I. C., Saxena, R., Scheer, F. A. J. L. & Rueda, C. CIRCUST: A novel methodology for temporal order reconstruction of molecular rhythms; validation and application towards a daily rhythm gene expression atlas in humans. en. *PLOS Computational Biology* **19** (ed Hong, C. I.) e1011510. ISSN: 1553-7358. (2024) (Sept. 2023).

12. Liang, S., Wang, F., Han, J. & Chen, K. Latent periodic process inference from single-cell RNA-seq data. en. *Nature Communications* **11,** 1441. ISSN: 2041-1723. (2022) (Dec. 2020).

13. Leng, N., Chu, L.-F., Barry, C., Li, Y., Choi, J., Li, X., Jiang, P., Stewart, R. M., Thomson, J. A. & Kendziorski, C. Oscope identifies oscillatory genes in unsynchronized single-cell RNA-seq experiments. en. *Nature Methods* **12,** 947–950. ISSN: 1548-7091, 1548-7105. (2020) (Oct. 2015).

14. Karin, J., Bornfeld, Y. & Nitzan, M. scPrisma infers, filters and enhances topological signals in single-cell data using spectral template matching. en. *Nature Biotechnology* **41,** 1645–1654. ISSN: 1087-0156, 1546-1696. (2024) (Nov. 2023).

15. Wolf, F. A., Hamey, F. K., Plass, M., Solana, J., Dahlin, J. S., Göttgens, B., Rajewsky, N., Simon, L. & Theis, F. J. PAGA: graph abstraction reconciles clustering with trajectory inference through a topology preserving map of single cells. en. *Genome Biology* **20,** 59. ISSN: 1474-760X. (2024) (Dec. 2019).

16. Wang, S., McCormick, T. H. & Leek, J. T. Methods for correcting inference based on outcomes predicted by machine learning. en. *Proceedings of the National Academy of Sciences* **117,** 30266–30275. ISSN: 0027-8424, 1091-6490. (2023) (Dec. 2020).

17. Jolliffe, I. T., Trendafilov, N. T. & Uddin, M. A Modified Principal Component Technique Based on the LASSO. en. *Journal of Computational and Graphical Statistics* **12,** 531–547. ISSN: 1061-8600, 1537-2715. (2024) (Sept. 2003).

18. Hastie, T., Tibshirani, R. & Wainwright, M. *Statistical learning with Sparsity: the lasso and generalizations* eng. OCLC: 908931926. ISBN: 978-1-4987-1217-0 (CRC Press LLC, Boca Raton, 2015).

19. Wold, S. Cross-Validatory Estimation of the Number of Components in Factor and Principal Components Models. en. *Technometrics* **20,** 397–405. ISSN: 0040-1706, 1537-2723. (2024) (Nov. 1978).

20. Akbani, R. *et al.* A pan-cancer proteomic perspective on The Cancer Genome Atlas. eng. *Nature Communications* **5,** 3887. ISSN: 2041-1723 (May 2014).
