# Supplementary material for: Rhythm profiling using COFE reveals multi-omic circadian rhythms in human cancers in vivo
Source: PLoS Biol. 2025 May 27;23(5):e3003196. doi: 10.1371/journal.pbio.3003196 (PMC12136439; doi:10.1371/journal.pbio.3003196)
Supplement: Attachment — Submitted filename: PLoS_Bio_Response_to_Reviewers_auresp_3.pdf [file pbio.3003196.s022.pdf]

Response to Reviewers - PBIOLOGY-D-24-01923R2

Bharath Ananthasubramaniam and Ramji Venkataramanan

April 8, 2025

We thank the reviewers and the editor once again for their thoughtful input and help in improving our manuscript.

Response to Editor

In addition, we would like you to consider a suggestion to improve the title: "Rhythm profiling using COFE reveals multi-omic circadian rhythms in human cancers in vivo".

We accept the editor's suggestion for the title.

Response to Reviewers

Reviewer 2

1. Regarding the response to Major Comment #2: We acknowledge the authors' thorough explanation justifying the simplification of the optimization objective (Eq. 2 to Eq. 3) by dropping the cross-term related to orthogonality. The justification, based on the nature of sparse PCA where sparsity constraints often lead to near-orthogonality ($v_1^T v_2 \approx 0$), the empirical evidence showing the negligible contribution ($< 0.1\%$) of the term to the overall matrix approximation error, and the best solution selection, seems sufficient within the practical context of achieving sparsity via scPCA.

   However, upon examining Figure S1I, we note a potential trend where the fractional contribution of the neglected term appears to increase slightly with higher SNR. While the absolute magnitude remains very small, as stated by the authors, this relative trend warrants a brief mention. Thus, we suggest adding a brief cautionary note in the Methods or Discussion section to acknowledge this observation.

   We have now added a cautionary note in the 'Materials and methods' section as suggested that states 'Nevertheless, it is advisable to ensure that this matrix approximation error is small when applying scPCA to new datasets.' (Page 14)

2. Regarding the response to Major Comment #4: In response to Major Comment #4, the authors justified not adding an arbitrary threshold beyond the l_1 constraint itself by arguing (a) that the constraint inherently performs thresholding consistent with standard sparse PCA practice, and (b) that their cross-validation results show the optimal sparsity level is data-driven, with further thresholding increasing error and thus being suboptimal.

   However, our original question was not about suggesting that a different threshold for rhythmicity should be set. The comment extends beyond simply "how to determine the threshold" and poses the more fundamental question: "What is the justification for considering a non-zero value in the loading vector (v) as sufficient evidence for rhythmicity?" Therefore, an explanation is required as to how the mathematical condition $v_j = 0$ can represent the biological (or statistical) phenomenon

of 'rhythmicity' within the COFE model framework itself. We request the authors to elaborate on this fundamental link.

We now better understand the reviewer's question. There is a theoretical and applied answer to the question "What is the justification for considering a non-zero value in the loading vector (v) as sufficient evidence for rhythmicity?". We have added a new subsection to the 'Materials and methods' section on Page 14 titled "Identifying rhythmic features:" summarizing our answer.

The theoretical motivation is the close association between scPCA and cosinor analysis noted in S1 Text, where $\mathbf{v}_{1i}$ and $\mathbf{v}_{2i}$ correspond to the cosinor coefficients for the cosine and sine terms, respectively. Therefore, $\mathbf{v}_{1i} = \mathbf{v}_{2i} = 0$ is the definition of an arrhythmic feature. Rhythmicity testing can be formulated as model selection between "no rhythm" model (coefficients both zero) and "rhythm" (either coefficient is non-zero) as an alternative to the better known F-test [1]; see [2] and [3] for more on the model-selection based rhythm analysis. The asymptotic equivalence between model selection and cross-validation has been previously theoretically established [4, 5]. Therefore, the cross-validation procedure robustly chooses between the constant model ($\mathbf{v}_{1i} = \mathbf{v}_{2i} = 0$) and a rhythm model (non-zero $\mathbf{v}_{1i}$ or $\mathbf{v}_{2i}$) for each feature.

In practice too, COFE achieves perfect precision (no false discoveries) on synthetic data in almost all settings (S1G Fig.), consistent with our theoretical understanding of the workings of COFE.

One could of course consider setting further criteria to be more stringent in calling rhythmic features (e.g., setting an amplitude threshold on the recovered rhythms as we have recommended previously in our tool *compareRhythms* or even a higher threshold, $|\mathbf{v}_{1i}| > c$ or $|\mathbf{v}_{2i}| > c$, where $c > 0$). But in light of the very high precision already achieved, we found this further step unnecessary.

The relationship between statistically-significant and biological relevant rhythms is a broad and complex one and we have summarized our thoughts on this in [6].

References

[1] Cornelissen G. Applications of cosinor rhythmometry in pharmacology. Journal of Pharmacokinetics and Pharmacodynamics. 2021;48(3):339–359. doi:10.1007/s10928-021-09748-x.

[2] Weger BD, Gobet C, David FPA, Atger F, Martin E, Phillips NE, et al. Systematic analysis of differential rhythmic liver gene expression mediated by the circadian clock and feeding rhythms. Proceedings of the National Academy of Sciences. 2021;118(3):e2015803118. doi:10.1073/pnas.2015803118.

[3] Pelikan A, Herzel H, Kramer A, Ananthasubramaniam B. Venn diagram analysis overestimates the extent of circadian rhythm reprogramming. The FEBS Journal. 2022;289(21):6605–6621. doi:10.1111/febs.16095.

[4] Stone M. An Asymptotic Equivalence of Choice of Model by Cross-Validation and Akaike's Criterion. Journal of the Royal Statistical Society Series B (Methodological). 1977;39(1):44–47.

[5] Hastie T, Tibshirani R, Friedman J. The Elements of Statistical Learning. Springer Series in Statistics. New York, NY: Springer New York; 2009. Available from: http://link.springer.com/10.1007/978-0-387-84858-7.

[6] Ananthasubramaniam B. Are We Finding Functional or Merely Statistically Significant Rhythms? Journal of Biological Rhythms. 2023;38(6):535–536. doi:10.1177/07487304231194663.
